# PAF‐induced inflammatory and immuno‐allergic ophthalmic diseases and their mitigation with PAF receptor antagonists: Cell and nuclear effects

**DOI:** 10.1002/biof.1848

**Published:** 2022-05-20

**Authors:** Najam A. Sharif

**Affiliations:** ^1^ Singapore Eye Research Institute (SERI) Singapore Singapore; ^2^ Department of Pharmacology and Neuroscience University of North Texas Health Sciences Center Fort Worth Texas USA; ^3^ Department of Pharmacy Sciences Creighton University Omaha Nebraska USA; ^4^ Department of Pharmaceutical Sciences, College of Pharmacy and Health Sciences Texas Southern University Houston Texas USA; ^5^ Department of Surgery & Cancer Imperial College of Science and Technology London UK; ^6^ Duke‐National University of Singapore Medical School SingHealth Singapore Singapore

**Keywords:** allergic conjunctivitis, histamine, mast cell mediators, mast cells, nuclear receptors, ocular itch, PAF, PAF antagonists

## Abstract

Ocular allergies are becoming more prevalent as more airborne pollutants, irritants and microbes pervade our environment. Inflammatory and allergic mediators released by dendritic and mast cells within the conjunctiva cause allergic conjunctivitis (AC), a prevalent ocular surface disorder that affects >40% of the world's human population on a seasonal or perennial basis. Even though histamine is a major culprit, platelet‐activating factor (PAF) also contributes to AC, acting either directly or synergistically with histamine and other mediators. PAF receptor‐meditated inflammatory reactions, via cell‐membrane‐bound and nuclear‐membrane‐bound and nuclear PAF receptors, are also implicated in the etiology of other eye diseases such as uveitis, diabetic retinopathy, corneal and choroidal neovascularization, and age‐related macular degeneration which cause serious visual impairment and can lead to blindness. This review highlights the various deleterious elements implicated in the pathological aspects of ocular allergic reactions and inflammation and provides concepts and treatment options to mitigate these eye disorders with a special focus on PAF and PAF receptor antagonists.

## 1 INTRODUCTION

**FIGURE 1:**
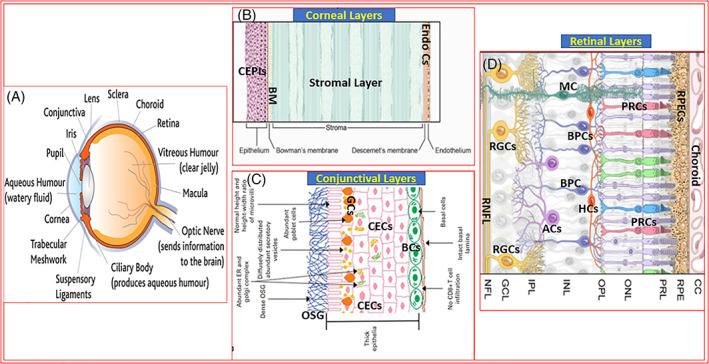
Overview of eye anatomy and the various layers and types of ocular cells associated with eye tissues. Figure 1A depicts the whole human and outlines the major structures and components. Figure 1B shows the layered structure of the cornea (BM, basement membrane; CEPIs, corneal epithelial cells; Endos Cs, endothelial cells). Figure 1C shows the cell types of the conjunctiva (BCs, basement cells; CECs, conjunctival epithelial cells; GCs, goblet cells; OSG, outer surface glycocalyx). Figure 1D illustrates the various specialized structures and cell‐types present in the retina (BPCs, bipolar cells; MC, Muller cell; PRCs, photoreceptor cells; RGC, retinal ganglion cell; RPEs, retinal pigmented epithelial cells; RNFL, retinal nerve fiber layer).

The eye is a highly sophisticated organ composed of a multitude of very specialized cells and tissues that subserve both structural and functional aspects of vision transmission to the brain for perception of the environment around us. Eyesight loss or even protracted visual dysfunctions are feared by most people, and hence protection and preservation of vision is important. Enhancing the quality of life of patients suffering from any ophthalmic disorder is an equally important goal for researchers and clinicians. As a prelude to the main topic of the review article, it is important to outline the basic anatomy of the eye (Figure 1A) with emphasis on the target tissues where platelet‐activating factor (PAF) elicits its pathological actions.

The eyeball is encased in a tough fibrous scleral tissue and the cornea at the front of the eye is in fact a specialized modification of the sclera and is totally transparent. The conjunctiva is an extension of the corneoscleral tissue and is highly vascularized unlike the cornea. Both the cornea and conjunctiva (Figure 1B,C) are composed of many layers of different types of cells. However, it is the epithelial cells of the cornea and conjunctiva which are impacted by the environmental radiation, irritant substances and allergens when they land on the ocular surface. The tear‐film that sits on the corneal and conjunctival epithelia provides oxygen, nutrients, anti‐oxidants and anti‐microbial agents to those cells, whereas the aqueous humor (AQH) in the anterior chamber (ANC) of the eye delivers the nutrients to the cells lining the ANC, including corneal endothelial cells, lens and iris epithelial cells, and the trabecular meshwork cells from where the AQH drains out. At the back of the eye, the choroidal blood circulation nourishes the retinal cells and removes their waste products. The retinal pigmented epithelial (RPE) cells and the outer basement membrane separates the photoreceptor cells from the choroidal blood vessels (Figure 1D).

The retina is extremely heterogenous in terms of the cell‐types present there and their diverse functions (Figure 1D). The light entering the cornea passes through the pupil and is focused on to the light‐sensitive photoreceptors (rods and cones) at the back of the eye where they lie adjacent to the RPE cells. The photoreceptor cells convert the light received into chemical messengers (neurotransmitters) which they release at their terminals close to the horizontal cells. The latter integrate the signals and in turn activate the bipolar and amacrine cells which then relay the information to the retinal ganglion cells (RGCs), again through neurotransmitters which activate their cognate postsynaptic receptors (Figure 1D). Finally, the RGCs collate and integrate all inputs and convert the information into electrical signals that are sent to the brain via the optic nerve for image processing and visual perception. Obviously, all these processes occur at sub‐second time‐scale.

## 2 PREVALENCE OF EYE DISEASES

Ophthalmic diseases represent an under‐appreciated public health issue. While they do not manifest as life‐and‐death scenarios, many millions suffer from a whole host of eye disorders that seriously affect quality of life and which contribute to global loss of productivity and represent a significant economic burden. The epidemiologic data on eye diseases and conditions collected by the World Health Organization (WHO)^1^ requires worldwide attention. Thus, by 2020 there were at least 2.6 billion people afflicted with myopia, >2 billion with presbyopia (poor near‐sight), 196 million with age‐related macular degeneration (AMD), > 150 million with diabetic retinopathy, and 76–80 million with glaucoma. It is estimated that >40% of the world population is adversely affected by some ocular surface symptoms associated with allergic conjunctivitis (AC). The involvement of PAF in the etiology of some of these diseases will be discussed in more detail ahead in this review.

## 3 PAF RECEPTOR SIGNALING AND PHYSIO‐PATHOLOGICAL ACTIONS OF PAF

**FIGURE 2:**
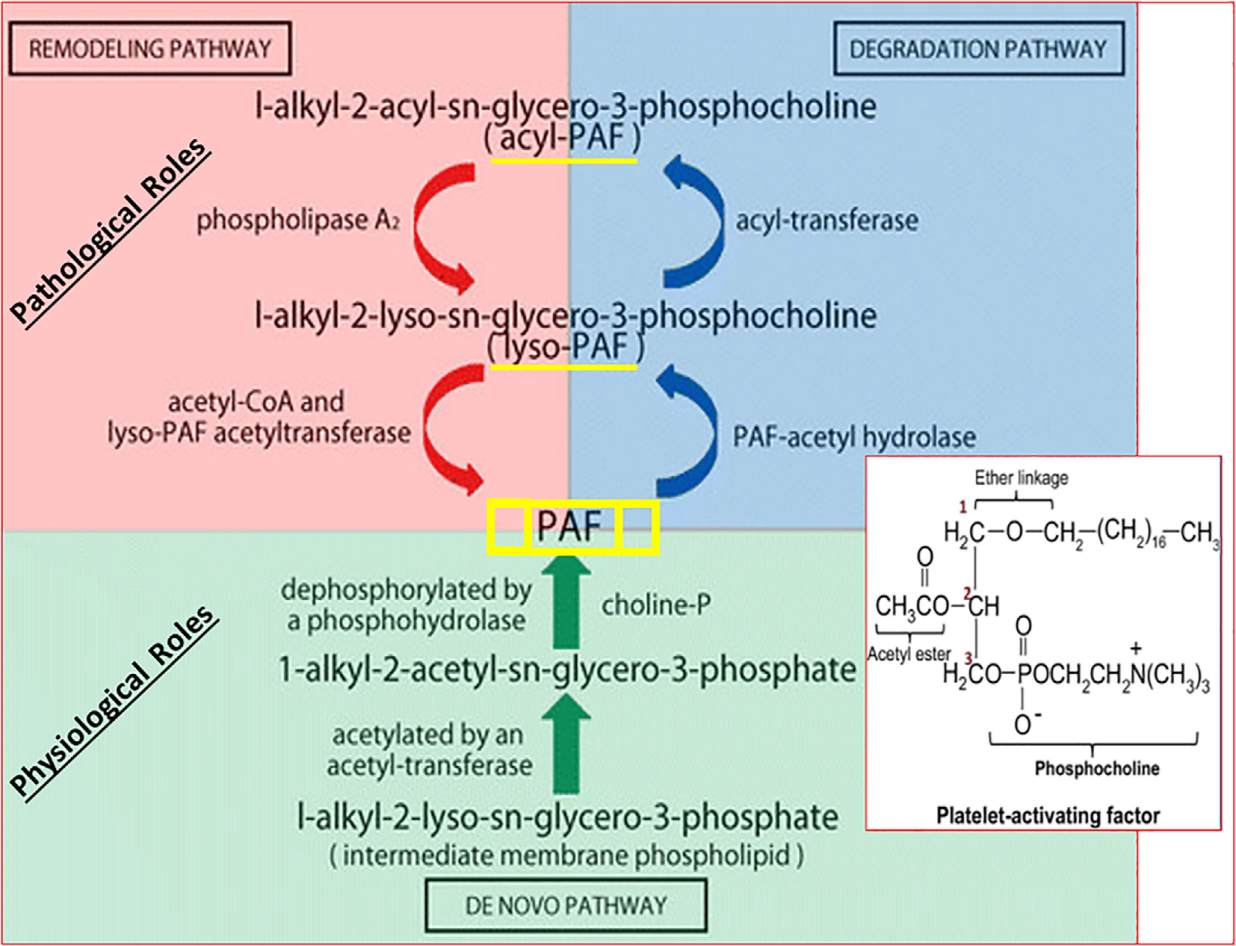
The synthetic and catabolic machinery for PAF as it may happen in mast cells and epithelial cells of the eye. Adapted and modified with gratitude from Reference 119.

The ether phospholipid, PAF, is generated from cell membrane phospholipids (Figure 2) and secreted into the extracellular space by numerous cell types including mast cells, basophils, eosinophils, neutrophils, and macrophages in response to local injury or allergen challenge. Indeed, different forms of PAF has been detected in normal and AC sufferers' tear‐film (C18:O‐PAF = 0.84 nM; C16:O‐PAF = 118 nM),^2^ and PAF is fairly rapidly metabolized to Lyso‐PAF once it has triggered and activated it signaling pathway components in the target cells.^2^, ^3^, ^4^, ^5^, ^6^, ^7^ Moreover, at least two splice variants of PAF receptors, differing in their ability to engage different cytoplasmic G‐proteins, are expressed by PAF‐sensitive cells. Some cells express only one form and others express both forms and yet the down‐stream signaling pathways may act in a synergistic manner.^2^, ^3^, ^4^, ^5^, ^6^, ^7^, ^8^

PAF exhibits a pico‐nano‐molar affinity and potency for its receptors with a pharmacological profile that is quite similar in the various bodily cells and tissues expressing PAF receptors. PAF is generated constitutively at femtomolar‐picomolar levels and such concentrations are maintained at a basal level to permit PAF to perform its physiological functions in most tissues and organs and help maintain homeostasis.^2^, ^3^, ^4^, ^5^, ^6^, ^7^, ^8^ PAF is somewhat unique since it exhibits autocrine, paracrine, juxtracrine (intracrine), or endocrine actions in the body and promotes cellular and neuronal health, long‐term potentiation, cell–cell signaling, cell proliferation and neurite growth.^5^, ^6^, ^7^ However, at higher sustained concentrations, PAF exhibits pathological features and thus causes many bodily dysfunctions resulting in several diseases including thrombosis, shock, asthma, and some central nervous system (CNS) disorders such as stroke, and has been implicated in ocular diseases (see ahead).^4^, ^5^, ^6^, ^7^

**FIGURE 3:**
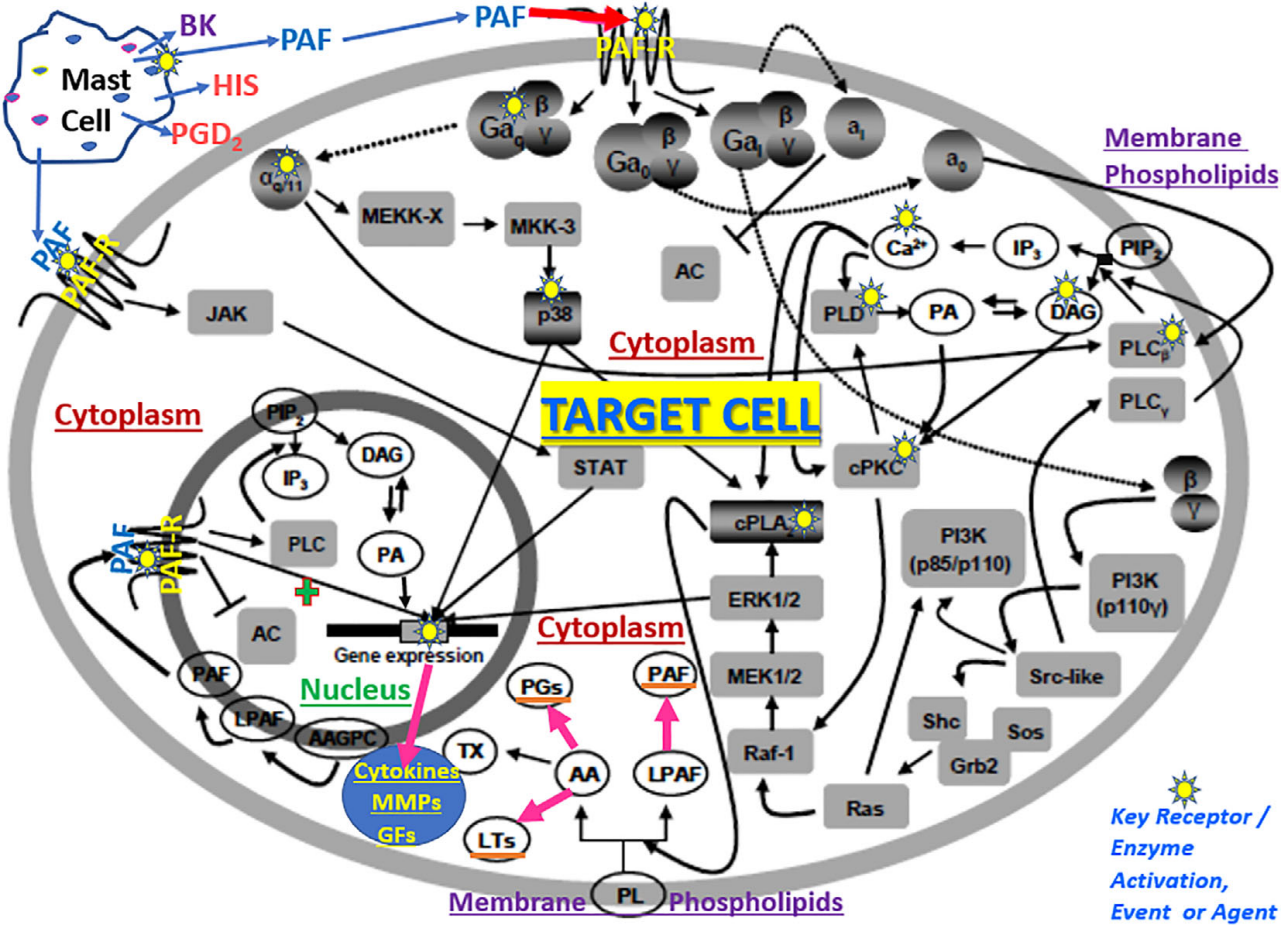
The signal transduction pathways associated with PAF receptors in the cell membrane, nuclear membrane and cytoplasmic compartments of PAF‐sensitive cells. Some of the most important abbreviated elements are as follows: AA, arachidonic acid; AC, adenylate cyclase; BK, bradykinin; DAG, diacyl glycerol; GFs, growth factors; HIS, histamine; IP3, inositol trisphosphate; MMPs, matrix metalloproteinases; PAF‐R, PAF receptor; PLC, phospholipase C; PLD, phospholipase D; PA, phosphatic acid; PL, phospholipid; PG, prostaglandin; LT, leukotriene; TX, thromboxane. Adapted and modified with gratitude from Reference 4.

The signal transduction pathways for PAF are complex involving phospholipid breakdown at the cell and nuclear membrane levels and involving cytoplasmic components and nuclear genetic transcription.^4^, ^5^, ^6^, ^7^ Thus, as shown in Figure 3, PAF receptor activation recruits Gα_0/q_‐protein which interacts with phospholipase C (PLC) which hydrolyzes phosphoinositides to generate the intracellular second messengers, inositol phosphates and diacylglycerol. The latter then mobilize intracellular Ca^2+^ ([Ca^2+^]_i_) and activate various protein kinases, respectively. The elevated [Ca^2+^]_i_ also activates cytoplasmic PLA_2_ to generate arachidonic acid from which prostaglandins (PGs) and LTs are produced, and the membrane‐bound phospholipid degradation yields intracellular PAF (Figure 3).^4^, ^5^, ^6^, ^7^ The latter binds to the nuclear membrane‐bound PAF receptors and triggers nuclear gene expression of cytokines, matrix metalloproteins (MMPs) and growth factors which are secreted by the activated cells (Figure 3).^4^ However, PAF‐receptor activation can simultaneously result in recruitment of Gα_i_‐protein to suppress adenylate cyclase activity and thus reduce intracellular levels of cAMP (Figure 3).^4^, ^5^, ^6^, ^7^

## 4 ROLE OF PAF IN OCULAR DISEASES

**FIGURE 4:**
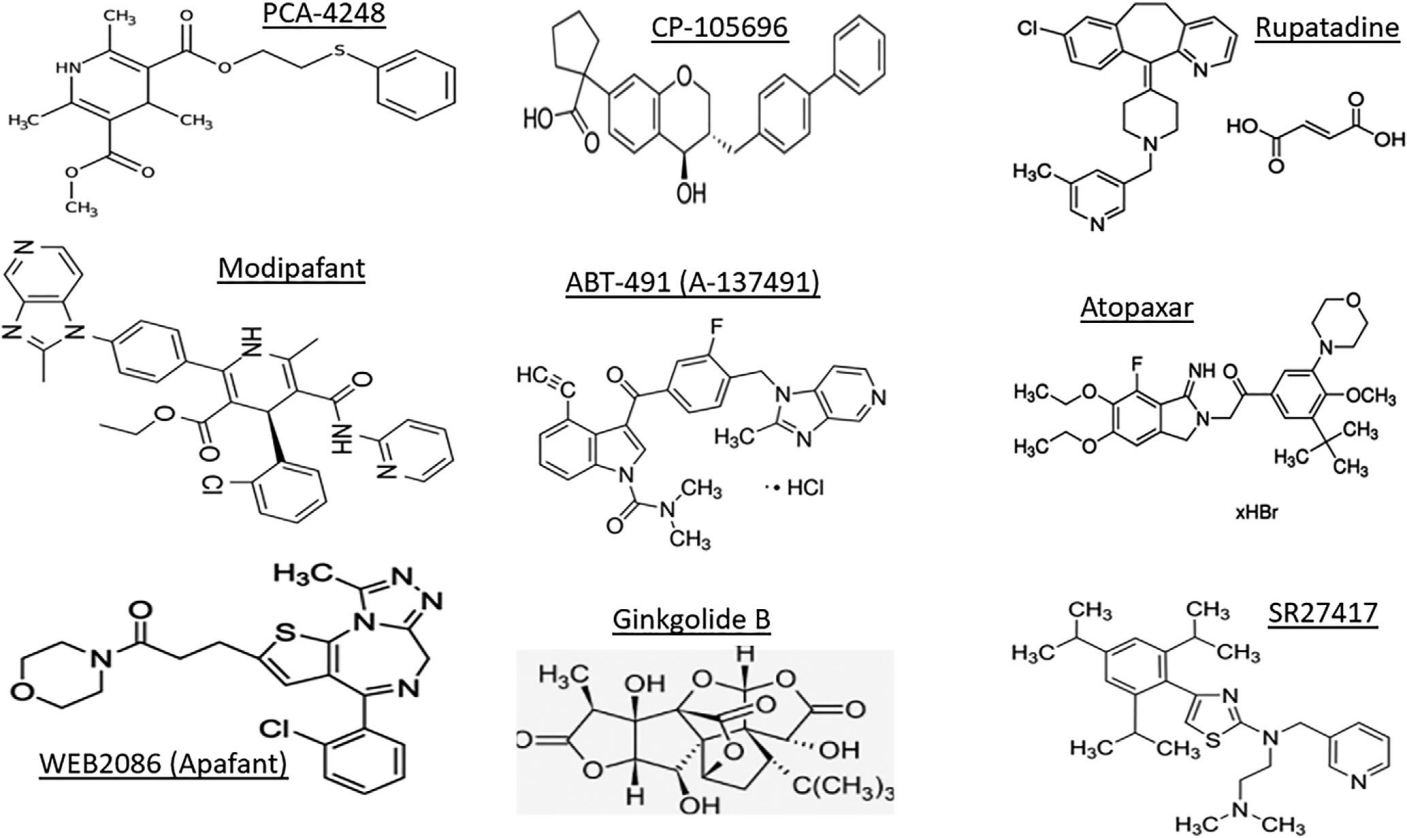
Structures of various PAF receptor antagonists of synthetic and natural origin.

**FIGURE 5:**
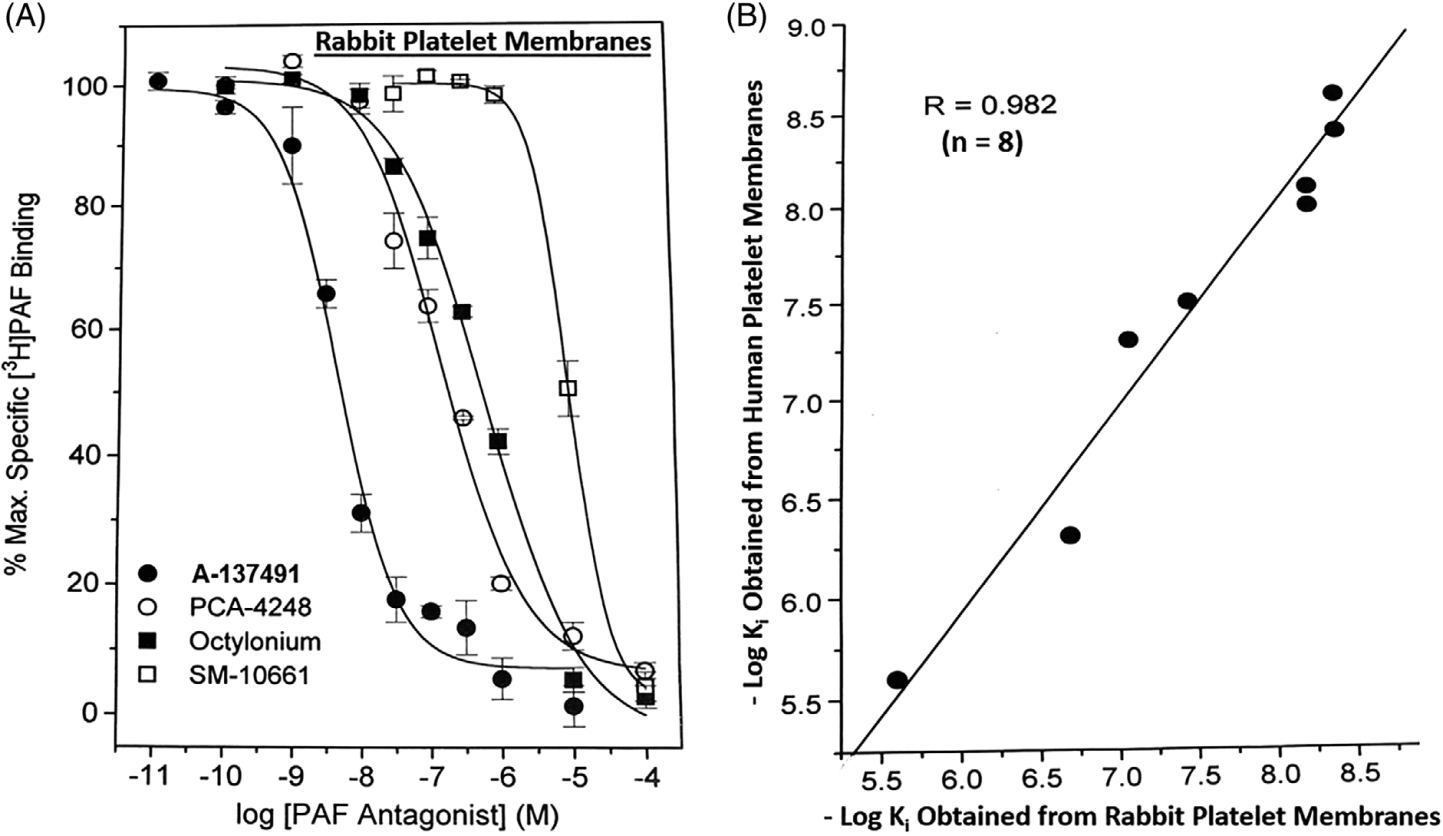
Pharmacological profile of PAR receptor binding to platelet membranes. Figure 5A depicts the concentration‐dependent inhibition by unlabeled PAF receptor antagonists of different affinities for [^3^H]‐PAF binding to rabbit platelet membranes in vitro. Figure 5B illustrates the correlation between the pharmacological profile of PAF receptor antagonists competing for [^3^H]‐PAF binding to rabbit and human platelet membranes.

As with HIS, PAF is a potent proinflammatory mediator that induces platelet aggregation, increasing vasodilation and blood vessel permeability, enhancing neutrophil and eosinophil migration, and inducing hyperemia, itching, pain and local edema. Consequently, PAF is responsible for many of the symptoms and structural changes associated with systemic and CNS diseases mentioned above and in ocular disorder like AC, diabetic retinopathy, and some aspects of corneal and choroidal neovascularization that can cause retinal detachment and macular degeneration. Indeed, in terms of specific ocular effects, PAF and its receptor mRNAs have been found in the cornea, iris, ciliary body, retinal ganglion cells, microglial cells, and in blood vessels of the choroid,^9^, ^10^ and is released into the tear film upon conjunctival provocation.^2^, ^3^, ^11^ Topically applied PAF causes conjunctival edema and raises intraocular pressure (IOP),^5^, ^6^, ^7^ and intracamerally injected PAF produces an inflammatory reaction with pronounced aqueous flare, corneal edema, and raises intraocular pressure, hallmark features of anterior uveitis.^12^ In addition, intracorneal injection of PAF causes a severe chemotactic response in the cornea and the surrounding conjunctiva. PAF that is exogenously added to cultured cells or rabbit corneal organ cultures induces gene expression of cyclooxygenase‐2,^13^, ^14^ plasminogen activator,^14^, ^15^ and matrix metalloproteinases (MMPs).^15^ Corneal injuries induce PAF generation as well and can result in corneal neovascularization that causes loss of vision in the affected eye(s).^16^, ^17^, ^18^, ^19^, ^20^, ^21^, ^22^, ^23^ Clearly, PAF receptor antagonists are indicated to prevent the down‐stream signaling and responses described above. Indeed, many different types of small molecules of diverse structures and origins (natural products and synthetic) have been discovered, characterized and validated as PAF receptor antagonists (Figures 4 and 5 and Tables 1 and 2).^8^, ^24^, ^25^, ^26^, ^27^, ^28^, ^29^, ^30^, ^31^

**TABLE 1:** Inhibition constants for various compounds generated against [^3^H]‐PAF receptor binding to rabbit platelet membranes.

Test compound	[^3^H]‐PAF receptor binding inhibition constant (*K*_*i*_, nM; Mean ± SEM)
Nupafant	1.7 ± 0.6
SR‐27415	2.9 ± 0.8
RO‐24‐4736	3.5 ± 0.4
PAF	3.7 ± 0.4
A‐137491	4.1 ± 0.1
(+)‐Modipafant (UK80067)	4.5 ± 0.8
TCV‐309	5.5 ± 0.2
YM‐264	5.1 ± 0.6
A‐85783	6.9 ± 1.0
E‐6123	9.3 ± 0.3
E‐5880	9.5 ± 0.5
(±)‐Modipafant (UK‐74505)	10.2 ± 1.7
CV‐6209	23.9 ± 6.7
BN‐50739	28.6 ± 8.4
BN‐50730 (Rocipafant) LAU‐8080	44.8 ± 7.2
Hexanolamine	164.3 ± 15.4
RO‐19‐3704	200.6 ± 22.5
Octylonium	378.1 ± 17.8
PCA‐4248	357.4 ± 45.0
BN‐52021(Ginkolide B)	378.4 ± 87.3
SCH‐37370 (Rupatadine)	381.7 ± 152.9
CV‐3988	2902 ± 240
Etizolam	2321 ± 1358
SM‐10661	7493 ± 875
MN‐10021	31,107 ± 8750
RP‐55778	32,739 ± 11,655

**TABLE 2:** [^3^H]‐PAF and [^3^H]‐WEB‐2086 binding inhibition constants obtained from human platelet membranes.

Test compound	[^3^H]‐PAF receptor binding inhibition constant (*K*_*i*_, nM; Mean ± SEM)	[^3^H]‐WEB‐2086 binding inhibition constant (*K*_*i*_, nM; Mean ± SEM)
SR‐27415	2.6 ± 0.3	nd
PAF	3.1 ± 0.6	26.5 ± 2.0
A‐137491	3.9 ± 0.5	nd
YM‐264	7.3 ± 1.8	nd
TCV‐309	9.4 ± 3.1	nd
BN‐50739	53.4 ± 1.9	nd
CV‐6209	214.6 ± 12.9	66.3 ± 11.3
PCA‐4248	634	nd
CV‐3988	3090 ± 889	3783 ± 759
Octylonium	4335 ± 17.8	1820 ± 17.8
SM‐10661	22,756 ± 449	9850 ± 1353

Abbreviation: nd, not determined.

### 4.1 Allergic conjunctivitis

As the world population continues to rise and as under‐developed nations embrace the industrial and technological revolutions, there is a concomitant increase in worldwide pollution, both man‐made and through natural causes such as soil erosion due to climatic changes. Consequently, our eyes are suffering more from eye‐strain due to increased computer use and from the dust and other airborne irritants and allergens including tree and shrub pollen, fungal spores and microbes. Even the inside environment poses issues since dust mites, pet dander and cosmetics cause ocular and respiratory problems. The resulting assault on our corneas and conjunctival tissues is triggering more eye allergies and the attendant discomfort, malaise, absenteeism, and loss in productivity. Comorbidities associated with nasal and respiratory irritation and allergic reactions are also increasing with a consequential increase in asthmatic conditions. Focusing on the eyes, many allergens landing on the ocular surface trigger allergic reactions that are classified as seasonal allergic conjunctivitis (SAC) or perennial allergic conjunctivitis (PAC) with additional forms of conjunctivitis categories.^32^, ^33^, ^34^, ^35^ Suffice it to say, the commonality amongst these ocular surface diseases is the individual and/or synergistic involvement of many potent inflammatory mast cell mediators which will be described and discussed below. It is important to remember that mast cells are very heterogeneous and many species differences and tissue‐specific effects have been reported.^36^, ^37^, ^38^, ^39^, ^40^

**FIGURE 6:**
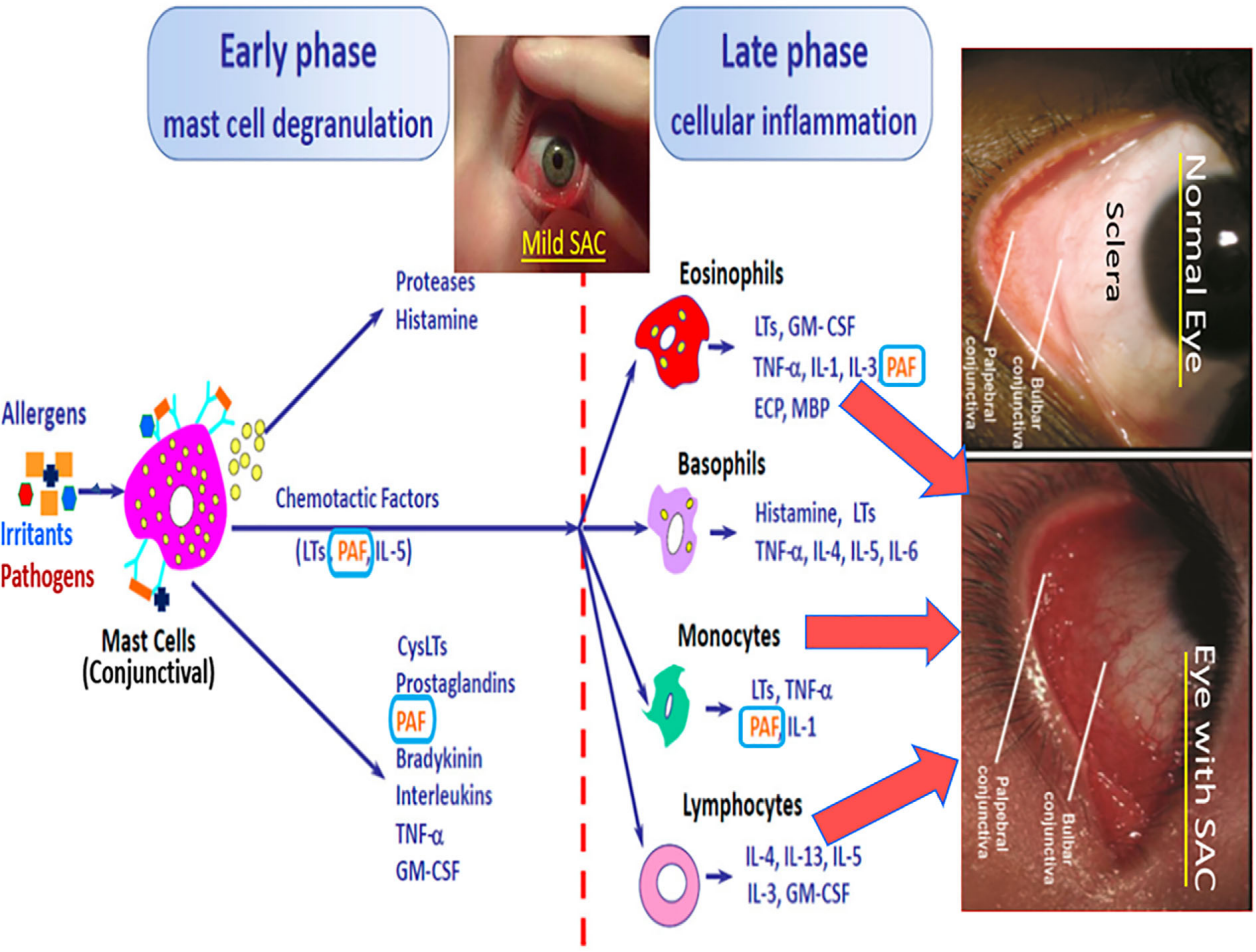
A schematic depiction of allergic conjunctivitis showing the relationship between mast cell mediators and their cellular influence and actions during the early‐ and late‐phases of the inflammatory/allergic reactions on the ocular surface of immunoglobulin‐sensitized patients. The insets depict mild and severe forms of AC compared with the normal ocular surface of the eye in human subjects. Adapted and modified with gratitude from Reference 79.

Even though regular blinking can potentially help rid of some of the allergens and irritants that enter the eye, once the latter come into contact with dendritic cells and B‐lymphocytes in the conjunctiva, they are cross‐linked to the immunoglobulin‐E (IgE) which binds to the high‐affinity IgE receptor on the mast cells. This results in the mast cells degranulating to release inflammatory mediators such as histamine (HIS), PAF, bradykinin (BK), a number of cytokines (interleukins; ILs; tumor neurosis factor‐α [TNF‐α];), prostaglandins (PGs), proteolytic enzymes (chymase and tryptase), and chemoattractants (IL‐5; leukotrienes [LTs]) into the tear‐film (Figure 6).^37^, ^38^, ^39^, ^40^ The small molecule mediators HIS/PAF/BK immediately vasodilate conjunctival blood vessels and increase their permeability. While this early‐phase inflammatory response acutely subsides over a few minutes. The next phase begins to develop and manifest when the mast cells and various immune cells (eosinophils, basophils, monocytes and lymphocytes) release more ILs, LTs, and cytokines to amplify the inflammatory allergic reaction as part of a delayed late‐phase secondary response to the allergen (Figure 6). The cytokines in turn induce IgE synthesis/release by B‐cells and cause inflammatory white blood cells (e.g., eosinophils) to infiltrate the conjunctiva, and to cause leukocyte migration, adhesion and activation. The signs and symptoms of AC are now obvious to the patient and the observers: red, itchy and swollen eyelids, and the patient seeks much needed relief from this irritating and bothersome affliction (Figure 6).^37^, ^38^, ^39^, ^40^ It is very likely that further amplification or sustaining of the latter AC condition occurs on the ocular surface since conjunctival epithelial cells (CECs), corneal epithelial (CEPI) cells and corneal fibroblasts (CFs) express receptors for many of the mast cell mediators and additive and synergistic actions between them prevail.^41^, ^42^, ^43^, ^44^, ^45^, ^46^, ^47^, ^48^, ^49^, ^50^, ^51^, ^52^ Even though HIS‐1 receptor antagonists, emedastine [Emadine]^53^, ^54^, ^55^, ^56^ or drugs possessing both HIS‐1 antagonist and mast cell stabilizing properties, olopatadine [Patanol® / Pataday® / Pazeo],^57^, ^58^, ^59^, ^60^, ^61^, ^62^, ^63^, ^64^ have clearly shown efficacy in combating many of the signs and symptoms of SAC/PAC and have been approved by health agencies for treatment of the latter disorders, there still is an unmet medical need for possible adjunctive therapeutics directed at other conjunctival mast cell mediators such as LTs, BK, and especially PAF.^65^, ^66^, ^67^, ^68^, ^69^, ^70^ Accumulated evidence strongly supports such a notion as will be discussed below.

#### 4.1.1 PAF receptors and actions in human conjunctiva related to AC

**FIGURE 7:**
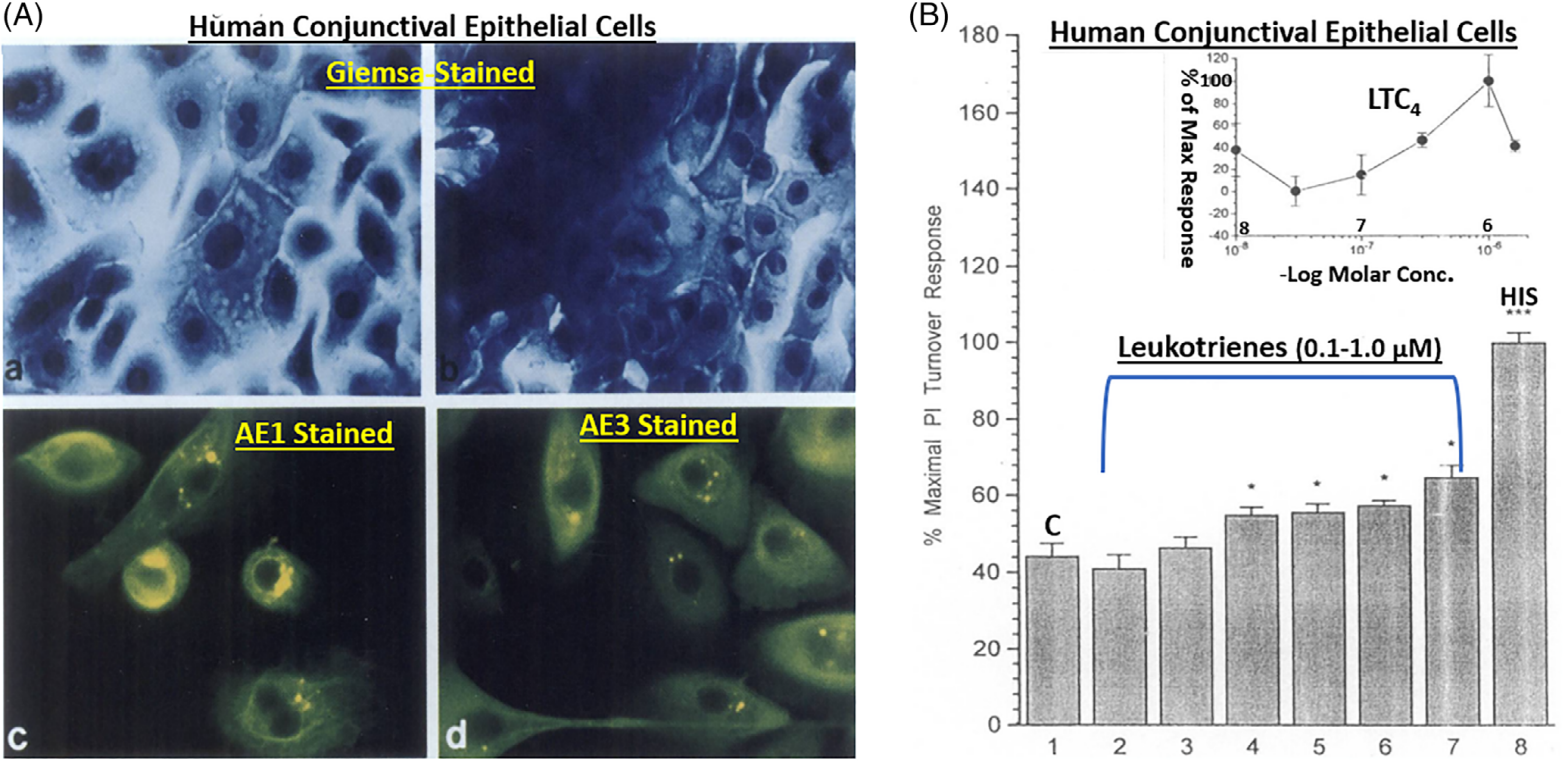
The morphological and immunohistochemical verification of human conjunctival epithelial cells (CECs) and their functional responses to various mast cell mediators. Figure 1A shows how the CEC staining with certain markers of epithelial cells helped qualify the isolated human CECs. Figure 1B depicts the ability of histamine (HIS) and various leukotrienes (LTs) to stimulate the production of [^3^H]‐inositol phosphates in human CECs after phosphoinositide (PI) hydrolysis relative to baseline control (C). Many other mast cell mediators were also tested in these cells (e.g., Figure 8A; References 45, 46). Adapted and modified with gratitude from References 45, 46. The astrisk symbols denote p < 0.05 ‐ 0.01.

**FIGURE 8:**
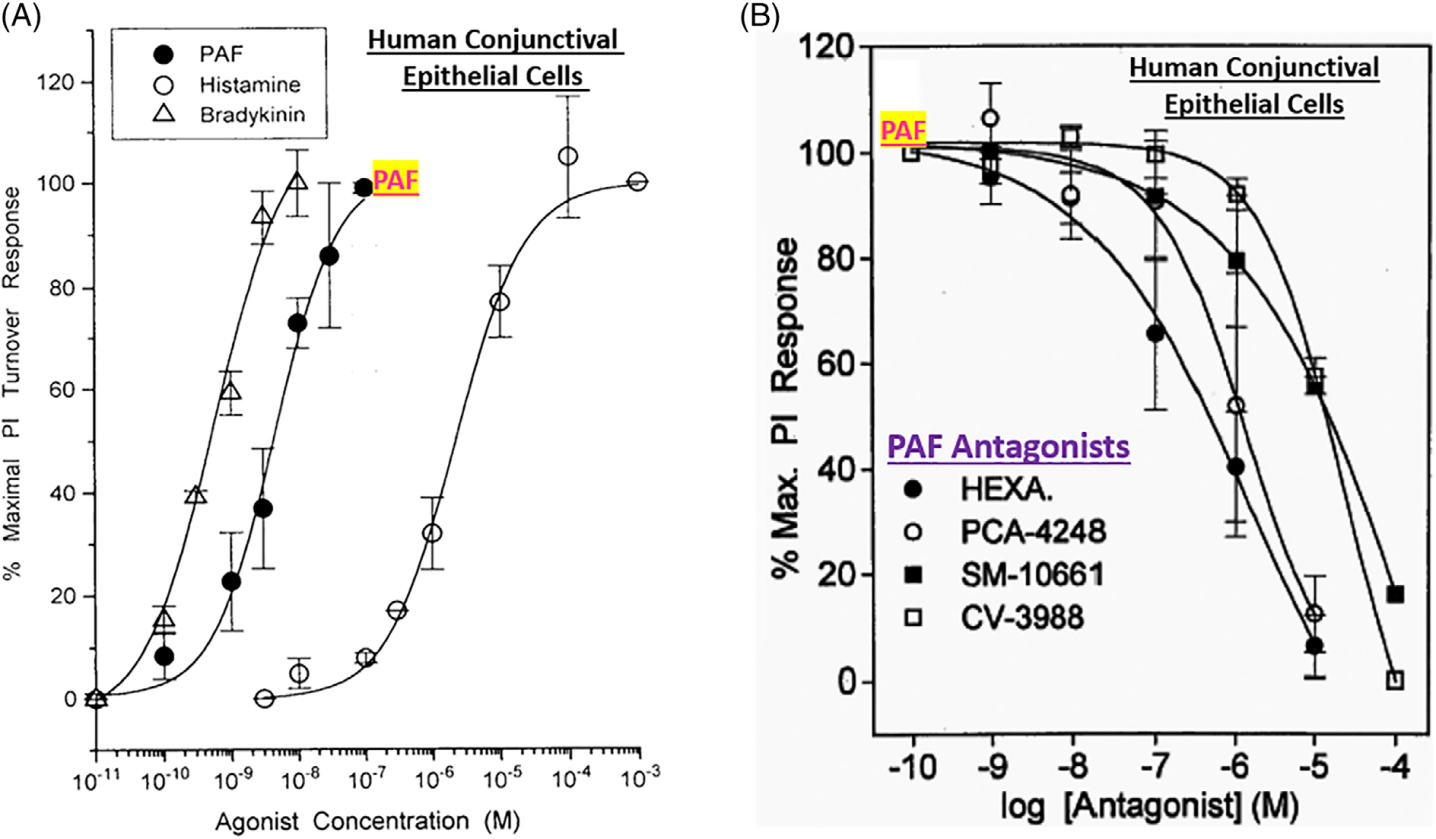
The concentration‐dependent activation of bradykinin, PAF and histamine receptors by their cognate ligand agonists in isolated human conjunctival epithelial cells is shown in Figure 8A. The ability of various concentrations of PAF receptor antagonists to block PAF‐induced functional responses in these cells is shown in Figure 8B. See References 45, 46, 65 for more information. Adapted and modified with gratitude from References 45, 46, 65.

**FIGURE 9:**
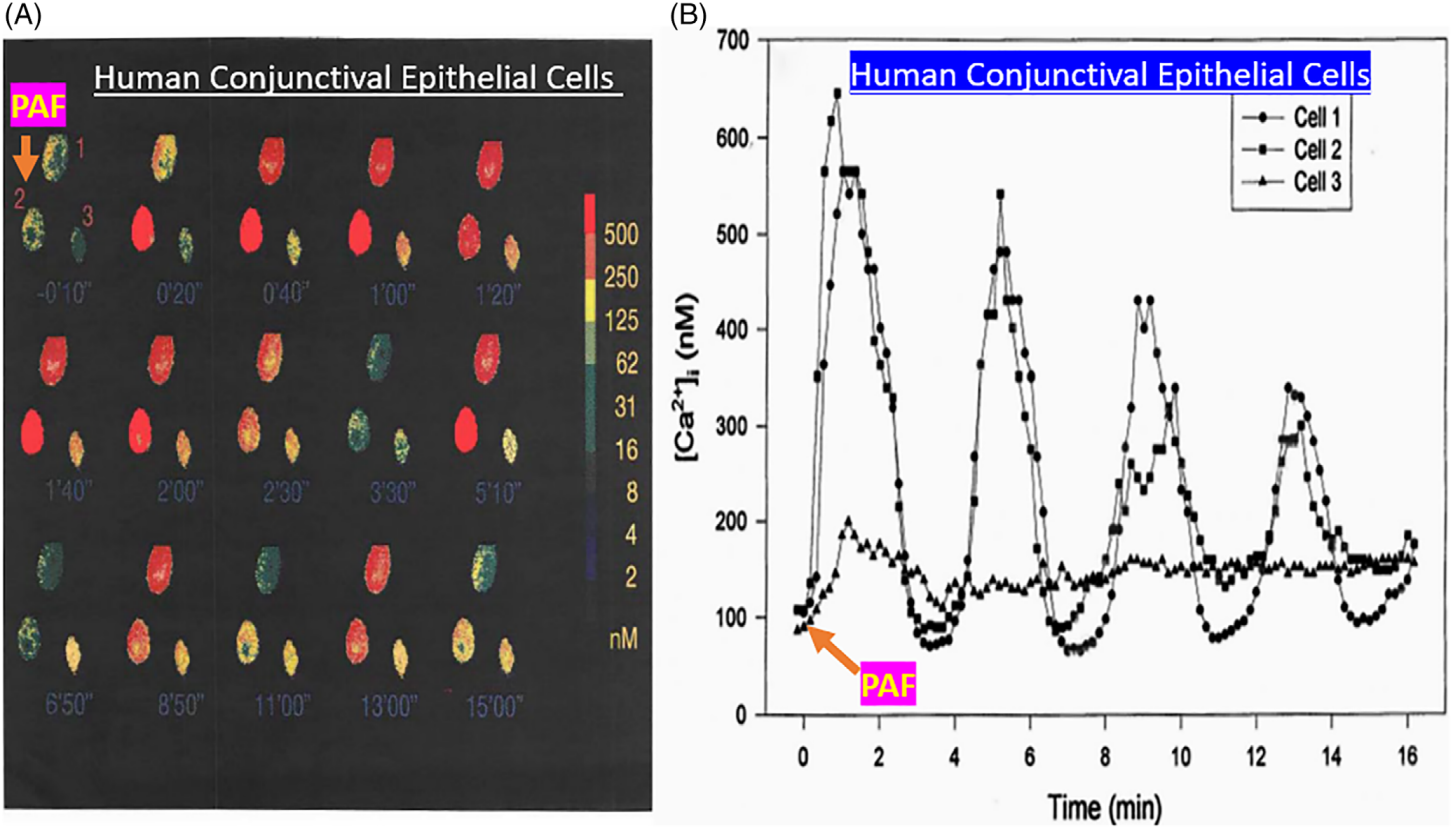
The PAF‐induced mobilization of [Ca^2+^]_i_ in isolated human conjunctival epithelial cells in real‐time (in seconds) is shown in adjacent cells in Figure 9A. The generation of [Ca^2+^]_i_ waves appears coordinated in an intracrine manner once PAF stimulates the cells (Figure 9B). See Reference 65 for more information. Adapted and modified with gratitude from Reference 65.

**FIGURE 10:**
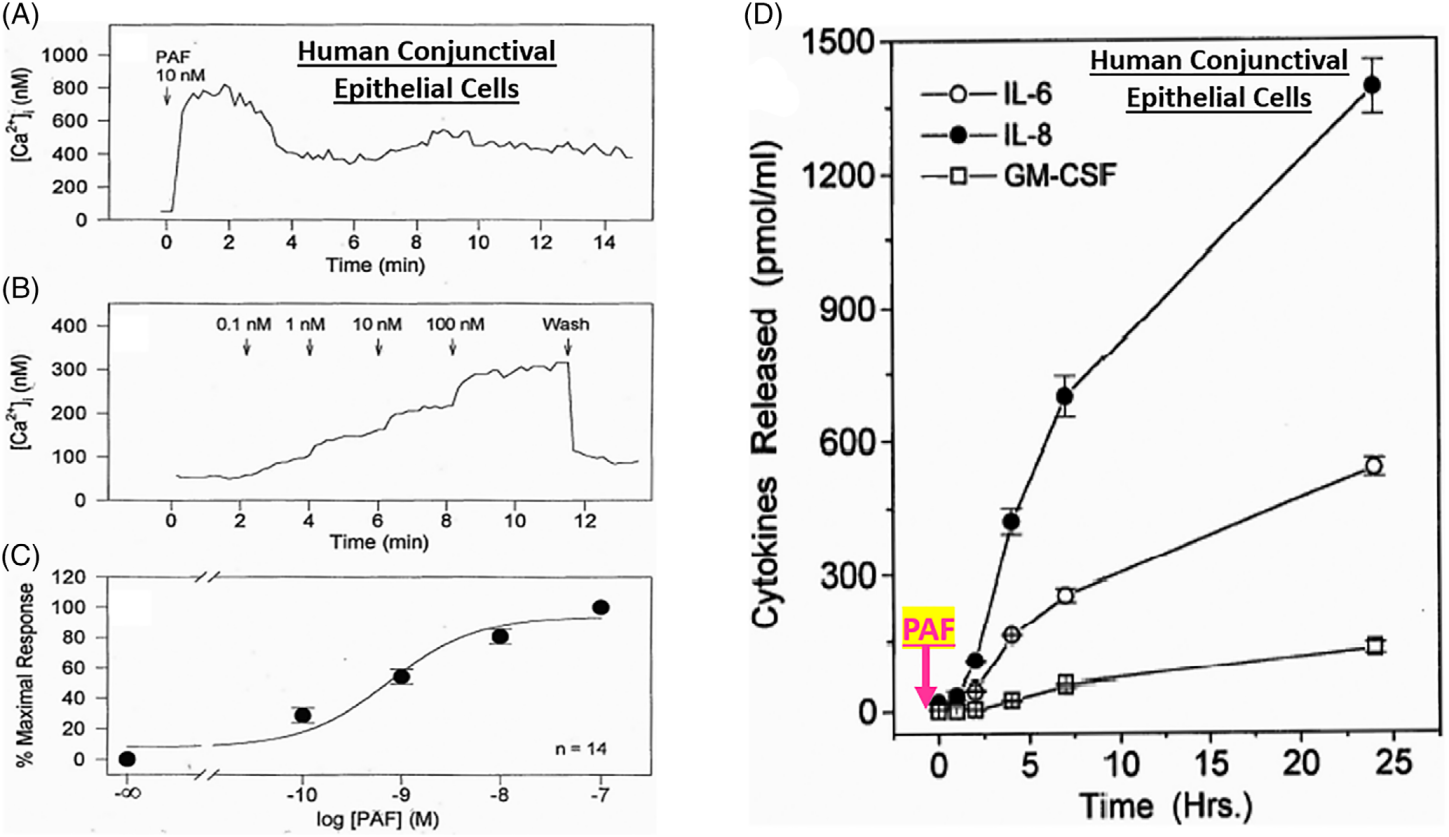
The concentration‐dependent stimulatory effect of PAF on [Ca^2+^]_i_ mobilization in different batches of isolated human conjunctival epithelial cells is shown in Figures 10A–C. The net result of such PAF receptor activation in these cells over many hours (simulating the late‐phase of allergic conjunctivitis) is the generation of various inflammatory cytokines with the highest amount being of IL‐8 > IL‐6 ≫ GM‐CSF (granulocyte macrophage colony stimulating factor) over a 24‐h period. See Reference 65 for more information. Adapted and modified with gratitude from Reference 65.

**FIGURE 11:**
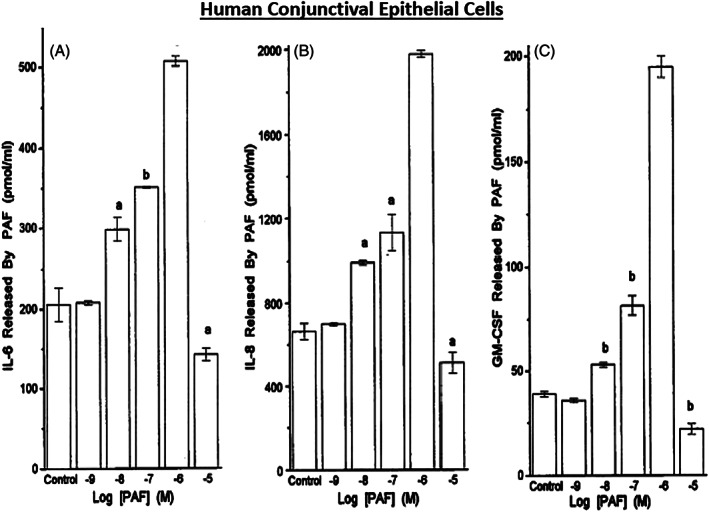
The concentration‐dependent stimulatory effect of PAF on IL‐8, IL‐6, and GM‐CSF production and release into the extracellular medium from isolated human conjunctival epithelial cells is shown. See Reference 65 for more information. Adapted and modified with gratitude from Reference 65. The letters a and b denote p < 0.05 ‐ 0.01 relative to control.

**FIGURE 12:**
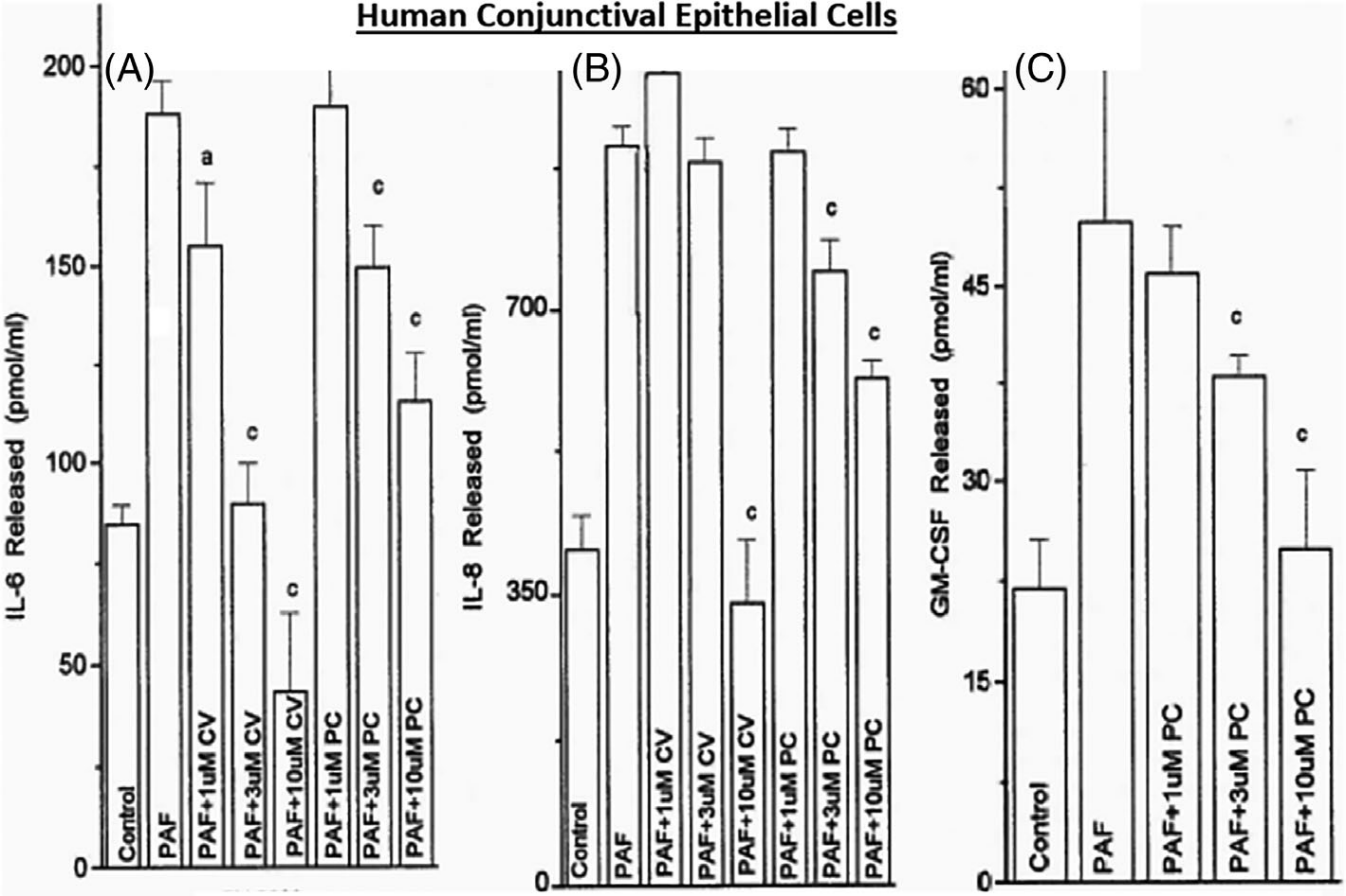
The ability of different concentrations of two PAF receptor antagonists (PC‐4248 and CV‐3988) to block the PAF‐induced cytokine secretion from isolated human conjunctival epithelial cells is shown. See Reference 65 for more information. Adapted and modified with gratitude from Reference 65. CV (CV‐3988) and PC (PCA‐4248) are PAF antagonists identified earlier in the Figures and text. The letters a and c denote p < 0.05 and p < 0.01, respectively.

In order to better understand the range of receptors present on immunohistochemically characterized (Figure 7A) isolated human conjunctival epithelial cells (HCECs) responsive to various mast cell mediators, a survey was conducted using cAMP, [^3^H]‐inositol phosphates ([^3^H]‐IPs]) and intracellular Ca^2+^ ([Ca^2+^]_i_) as readouts.^45^, ^46^, ^47^, ^48^ These studies clearly showed that HCECs expressed functionally active receptors for various LTs, HIS‐1, PAF and BK (Figures 7B and 8A) coupled to phosphoinositide (PI) hydrolysis which could be blocked by receptor‐selective antagonists (e.g., for PAF antagonists, Figure 8B).^45^, ^46^, ^47^, ^48^ Specifically, HCECs express PGE_2_ receptors and β2‐adrenoceptors which increased cAMP production, and BK, PAF, LTC4, and HIS receptors whose stimulation increased [^3^H]‐IPs (BK, EC_50_ = 0.83 nM; PAF, EC_50_ = 4.5 nM; LTC4, EC_50_ = 300 nM; HIS EC_50_ = 3.1 μM), and where substance P, endothelin and PGF_2α_ were inactive.^46^ [Ca^2+^]_i_ mobilization was also concentration‐dependently and potently induced by PAF (EC_50_ = 0.81 nM) which occurred in waves in adjacent HCECs (Figures 9 and 10A–C), a good example of cell–cell communication in real‐time, which was also antagonized by PAF‐receptor antagonists, CV‐3988 and PCA‐4248.^65^ Importantly, HCECs exposed to different concentrations of PAF responded by enhancing the synthesis and secretion of numerous inflammatory cytokines such as IL‐6, IL‐8, and GM‐CSF in a time‐dependent and concentration‐dependent manner (Figures 10D and 11), actions that were also curtailed by CV‐3988 and PCA‐4248 (Figure 12).^65^ These cytokines participate in triggering of the late‐phase AC and cause chemosis of T‐lymphocytes, basophils and neutrophils and the enhance adhesion molecules expression, secretion of proinflammatory enzymes, and to induce additional cytokine release in a vicious cyclic manner.^66^, ^67^, ^68^ Moreover, PAF triggers eosinophil accumulation and elevates expression of PAF receptors, which then enhances vascular permeability, edema, and ocular itching.^51^, ^69^ These properties of PAF are highly correlated with the signs and symptoms of AC, and thus mast cell‐derived PAF most likely has additive and/or synergistic effects with HIS in the initiation and progression of AC. Consequently, SAC/PAC treatment regimen would benefit from combining HIS‐ and PAF‐receptor antagonists in order to take advantage of possible additive and/or synergistic efficacy against these disorders.

#### 4.1.2 PAF receptors in human corneal epithelium and their role in AC and corneal injuries

**FIGURE 13:**
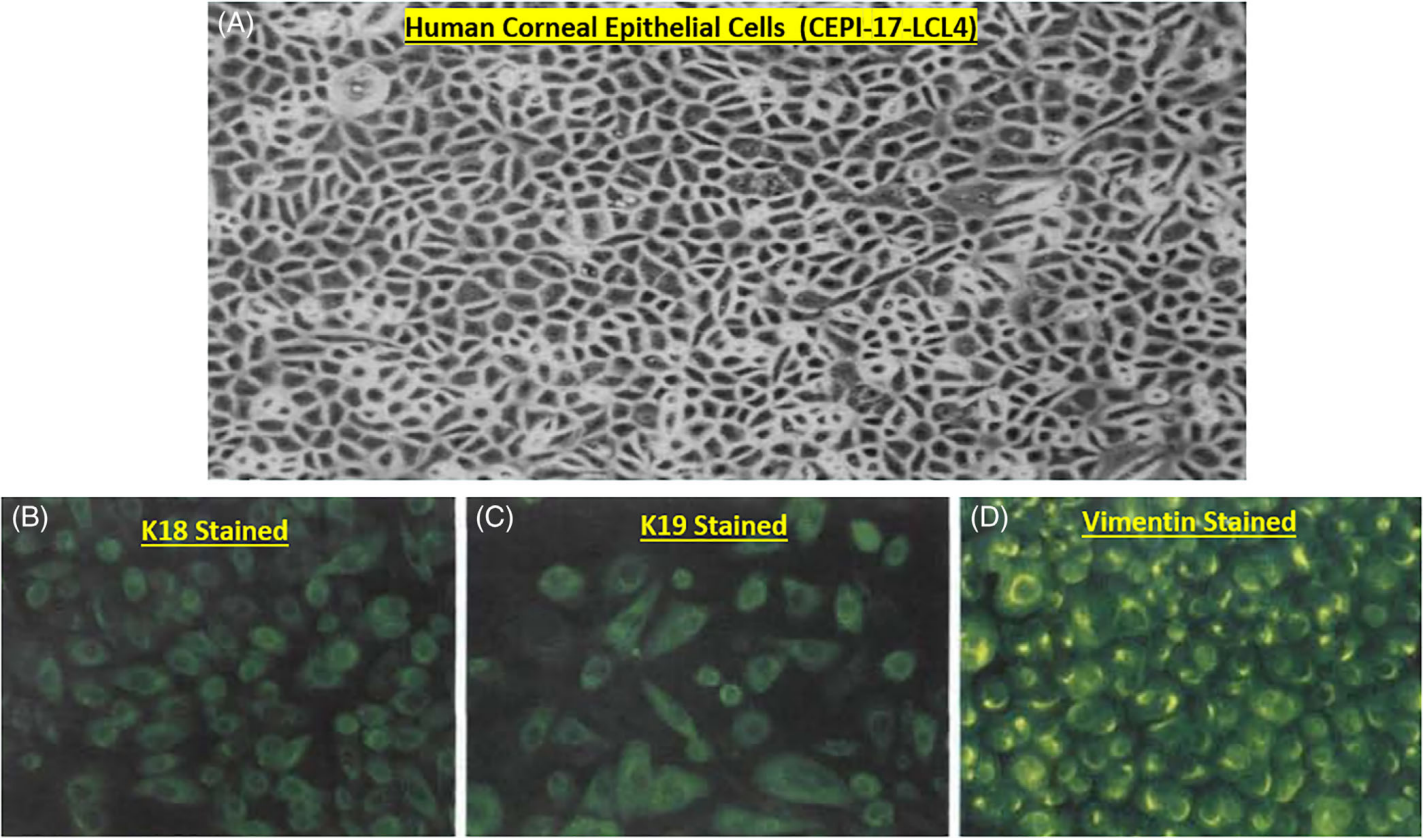
The morphological profile (Figure 13A) and immunohistochemical verification of the epithelial cell nature of immortalized human corneal epithelial cells are illustrated (Figures 13B–D). These cells were shown to be very similar to normal CEPI cells in terms of their morphology and cellular responses to various mast cell mediators and the antagonism of such functional responses to the cognate receptor antagonists (see Figure 14 and References 70, 71, 72, 73, 74 for more information). Adapted and modified with gratitude from References 14, 70, 71, 72, 73, 74.

**FIGURE 14:**
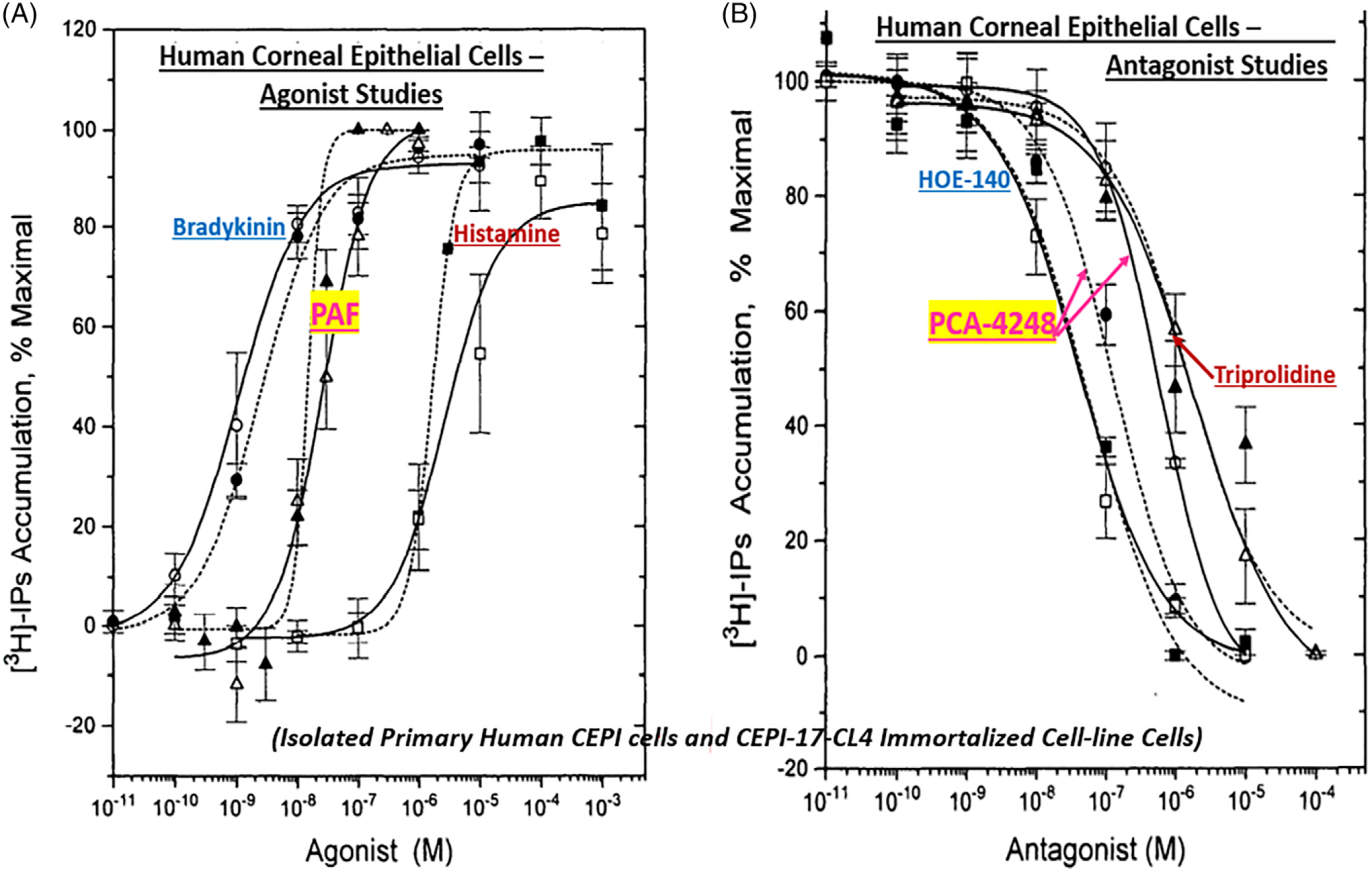
The concentration‐dependent functional activation of mast cell mediator receptors for bradykinin, PAF and histamine yielding accumulation of [^3^H]‐IPs in primary (normal) human corneal epithelial (CEPI) cells and immortalized cells (CEPI‐17‐CL4) are shown in Figure 13A. The concentration‐dependent blockade of such response by the respective receptor antagonists in both cell‐types are displayed in Figure 14B. Note the similarity of responses by the three agonists and their antagonists in both cell‐types indicating almost identical pharmacological profiles of these cells. See Reference 73 for more details. Adapted and modified with gratitude from Reference 73.

**FIGURE 15:**
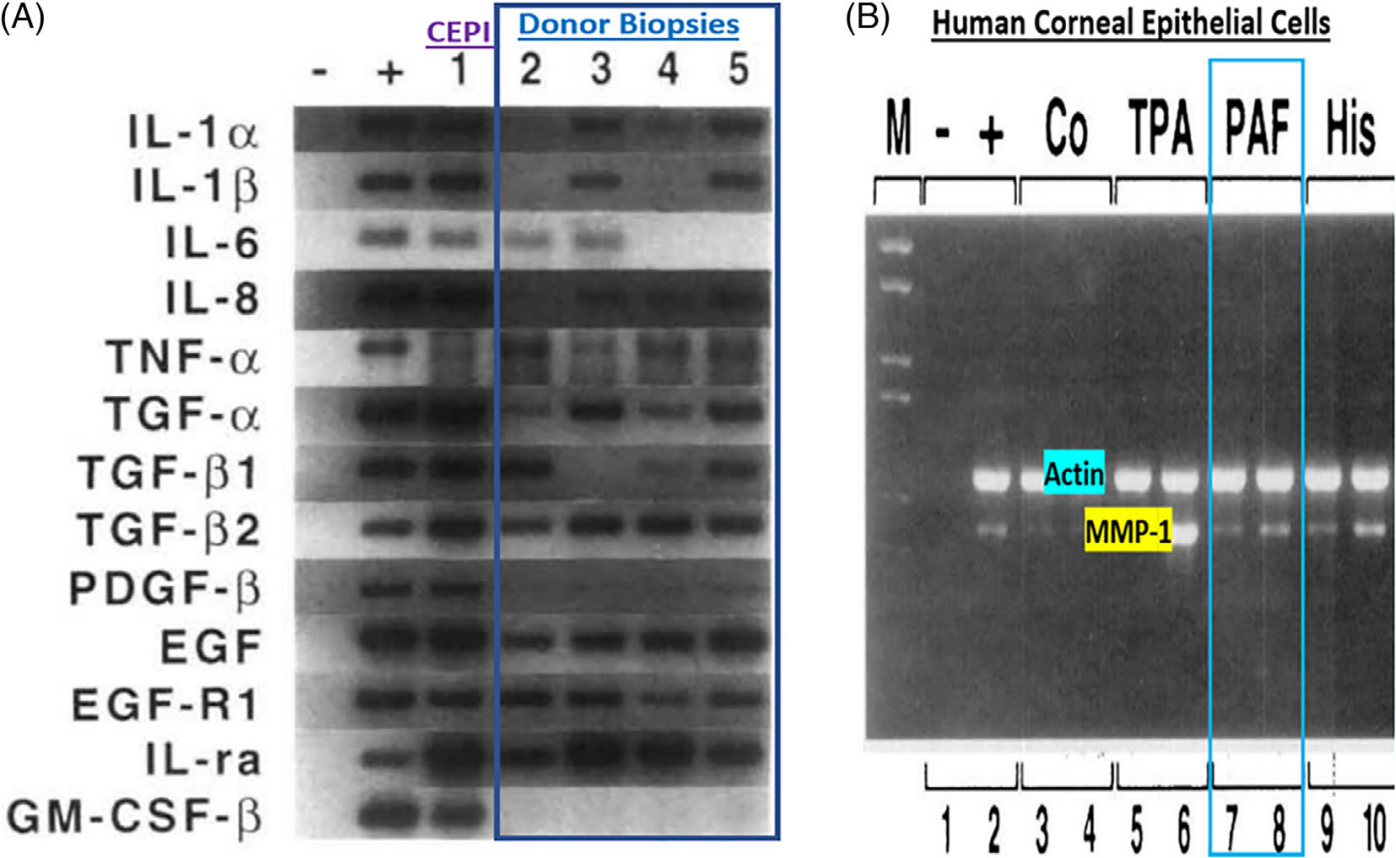
The presence of mRNAs for various inflammatory cytokines and growth factors within the corneal biopsies obtained from various human donors and in CEPI‐17‐CL4 cells are shown in Figure 15A (Reference 73). The up‐regulation of mRNA for matrix metalloproteinase‐1 (MMP‐1) in response to TPA (tetradecanoyl phorbol acetate), PAF and histamine (HIS) in CEPI‐17‐CL4 cells is displayed in Figure 15B. See Reference 70 for more details. Adapted and modified with gratitude from Reference 70.

**FIGURE 16:**
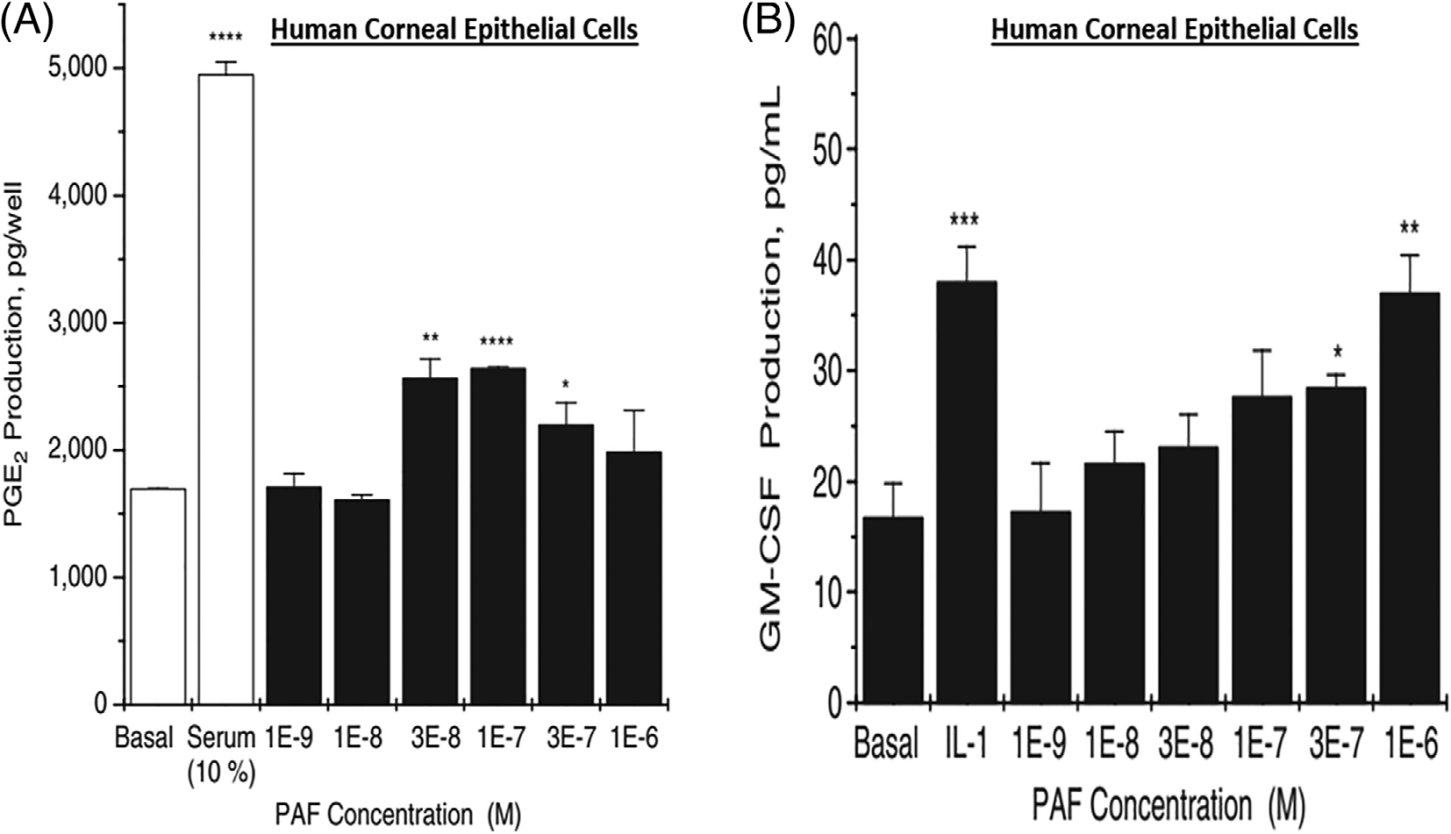
The production and release of prostaglandin E2 and the cytokine GM‐CSF from CEPI‐17‐CL4 cells in response to different concentrations of PAF are shown (Figure 16A: PGE_2_; Figure 16B: GM‐CSF). See Reference 4 for more information. Adapted and modified with gratitude from Reference 4. * p < 0.05; **p < 0.01; *** p < 0.001

**FIGURE 17:**
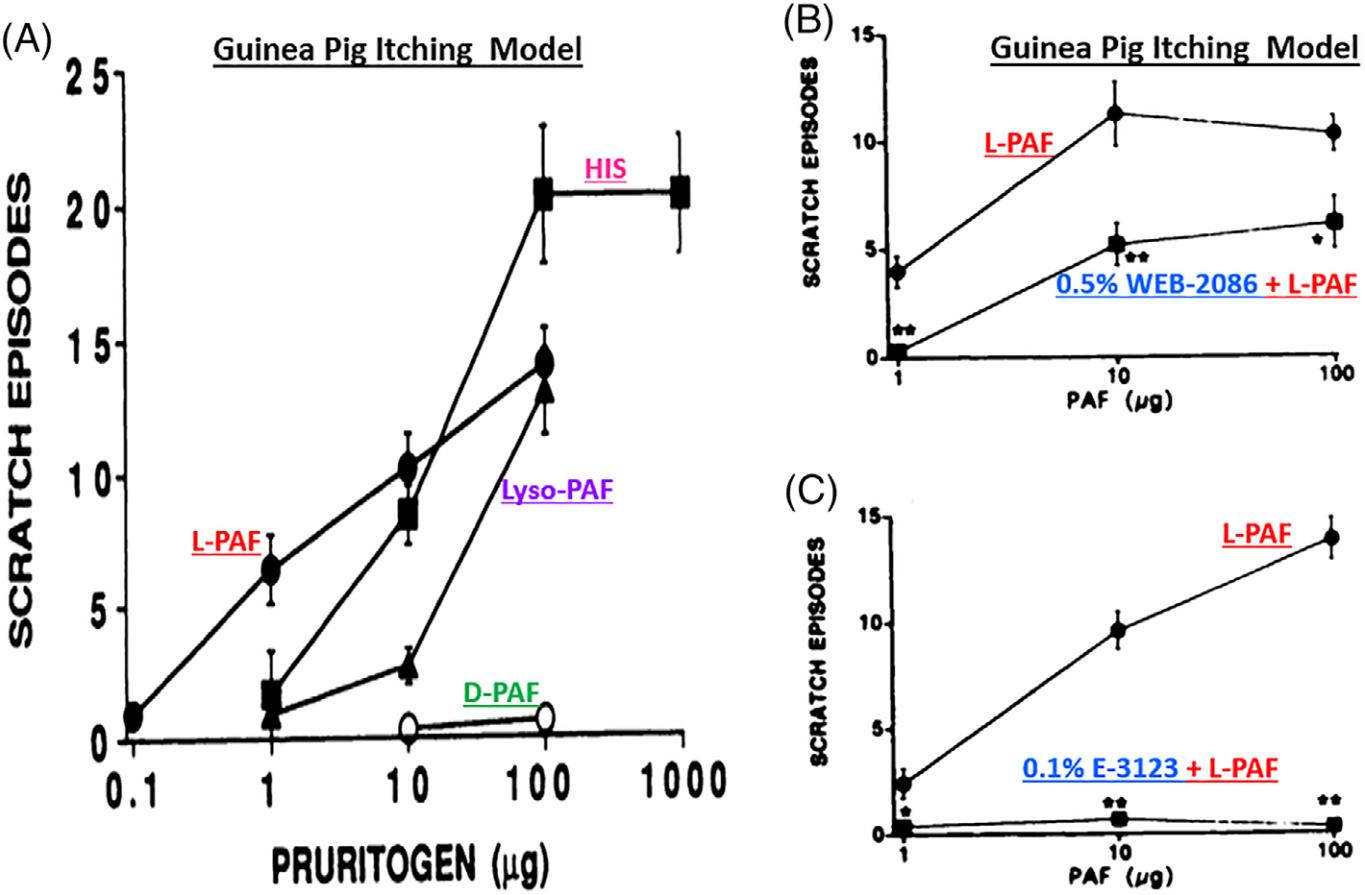
The scratching response of Guinea pigs representing ocular itch induced by topical ocularly applied L‐PAF, D‐PAF, Lyso‐PAF, and histamine (HIS) (Figure 17A), and the blockade of PAF‐induced responses by prior treatment with two different PAF receptor antagonists (Figures 17 B,C) are shown. Adapted and modified with gratitude from Reference 51.

**FIGURE 18:**
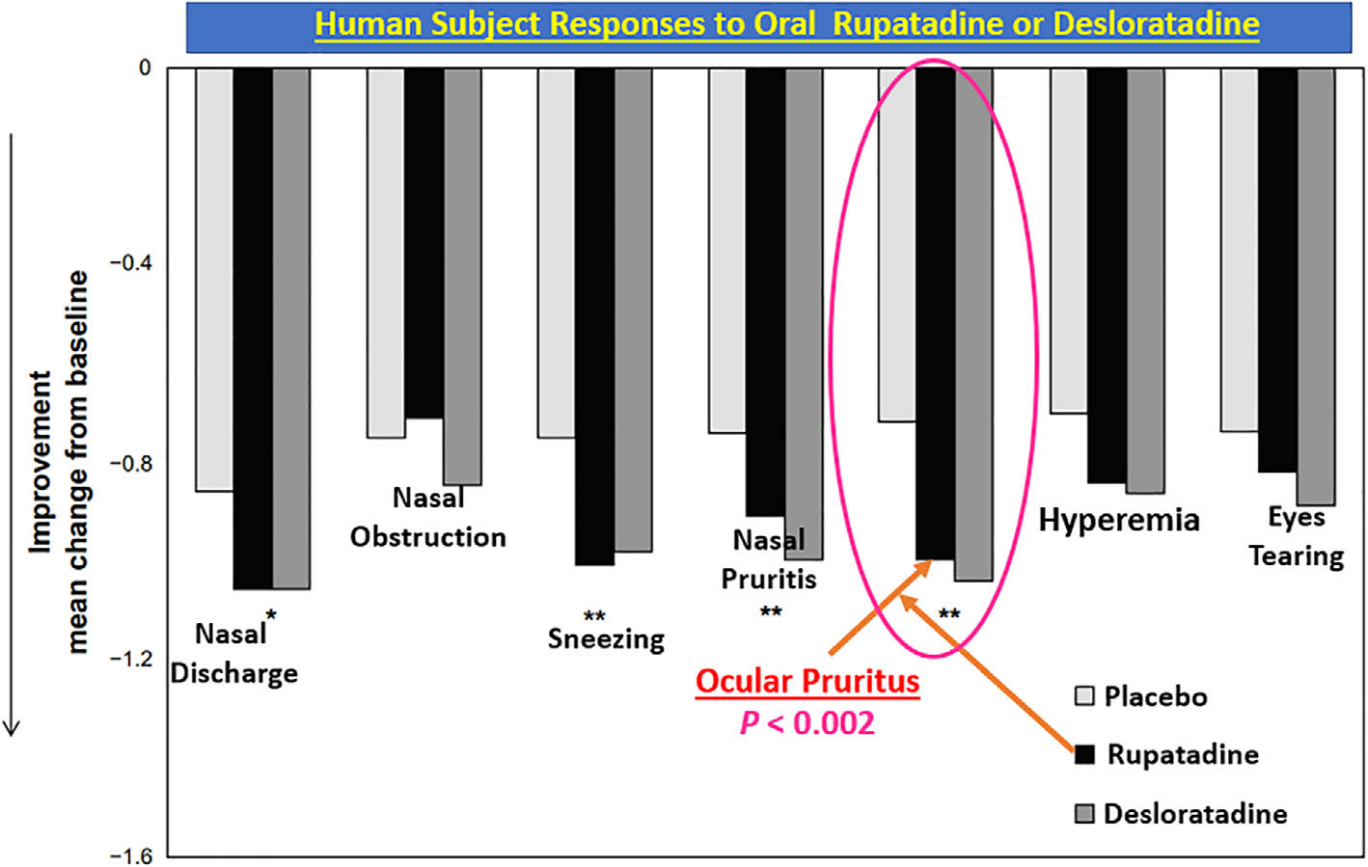
The ocular itching blockade by orally administered rupatadine, a PAF and histamine receptor antagonist, in patients suffering from rhino‐conjunctivitis is shown. Adapted and modified with gratitude from Reference 51.

Much of the early dogma on corneal functions centered on the transparency nature of the corneal epithelium permitting light to penetrate and reach the retinal tissue, and to a simple structural and thus a passive role. However, much research data now strongly supports a more dynamic role of the corneal epithelial cells in responding to mast cell mediators and acting synergistically with the epithelial cells of the conjunctiva.^42^, ^43^ Evidence for this hypothesis include the detection of nanomolar affinity PAF receptors in the bovine and rabbit cornea (*K*_*d*_ = 0.8–4.3 nM),^10^ similar to those found on rabbit and human platelets (*K*_*d*_ = 0.5 nM; Figure 5), and the ability of HIS, BK and PAF to induce functional responses in isolated and immunohistochemically characterized HCEPI cells and an immortalized cell‐line (CEPI‐17‐CL4; Figure 13), including PI turnover, [Ca^2+^]_i_ mobilization, cytokine and PG release, and intracellular expression of mRNAs for numerous growth factor and cytokines (Figures 14, 15, 16).^70^, ^71^, ^72^, ^73^, ^74^ Specifically, exogenously added PAF to HCEPI/CEPI‐17‐CL4 cells induced IPs production which was blocked by PCA‐4248 (Figure 14), MMP‐1 secretion (Figure 15), and GM‐CSF and PGE_2_ release (Figures 15 and 16).^70^, ^71^, ^72^, ^73^, ^74^ Such results independently verified and extended the earlier reports that PAF is a potent agent that helps remodel the cornea after injury or during and after initiation of AC.^13^, ^14^, ^15^, ^16^, ^17^, ^18^, ^19^, ^20^, ^21^, ^22^, ^23^, ^24^, ^75^, ^76^ The collective conclusions are that AC is an ocular inflammatory disorder in which multiple cells located in the conjunctiva and cornea interact and participate in the initiation and progression of the pathological condition, and where mast cell mediators, in particular HIS and PAF, trigger and maintain the acute and “chronic” (late‐phase) aspects of AC during seasonal changes in sensitized patients. Moreover, that the use of HIS antagonists, mast cell stabilizers and PAF receptor antagonists are strongly indicated, alone or in combination, to combat the signs and symptoms of AC, especially the itch associated with AC in which HIS and PAF are major culprits (Figure 17).^13^, ^14^, ^15^, ^16^, ^17^, ^18^, ^19^, ^20^, ^21^, ^22^, ^23^, ^24^, ^51^, ^67^, ^68^, ^69^ Importantly, oral rupatadine, a small molecule possessing micromolar affinities and potencies at both HIS and PAF receptors,^77^ and des‐loratadine (H_1_‐antagonist) not only significantly reduced nasal discharge, sneezing and nasal itching, but also significantly reduced the ocular itching (Figure 18) in patients suffering from rhinitis over a 4‐week period of dosing in multiple geographic locations in Europe.^78^, ^79^ Since both drugs were equally effective in reducing ocular itching, these data strongly support the notion that PAF receptor antagonists with a higher affinity and potency than rupatadine may offer a greater effectiveness in AC if dosed topical ocularly in sensitized SAC/PAC patients. Combination of health authority approved potent and effective HIS‐1 receptor antagonists such as emedastine and a future approved potent PAF antagonist would be expected to impart beneficial effects in combating SAC /PAC. Such studies are eagerly waited.

Since the ocular surface is exposed to the environment it is susceptible to injuries. It appears that PAF is a potent mediator of inflammation after corneal injuries, including alkali burns^13^, ^16^, ^18^, ^20^, ^21^, ^22^, ^23^, ^24^ where native resident PAF receptors mediate the responses.^9^, ^10^ Aside the inflammatory actions, PAF also releases vascular endothelial factor (VEGF)^20^, ^21^, ^22^, ^23^, ^24^ which is responsible for causing corneal neovascularization resulting in corneal opacity and serious visual impairment. Genetic deletion of PAF receptors resulted in suppressing aberrant new blood vessel growth over the cornea thereby confirming the role of PAF in this disorder.^21^, ^22^, ^23^ Fortuitously PAF receptor antagonists (e.g., CV‐3988 and Ginkgolide B) also inhibited PAF‐induced ocular surface neovascularization in naïve mice,^21^, ^23^, ^24^, ^25^, ^75^ and thus they can therapeutically help protect and repair the corneal architecture and prevent diminution of vision.

**FIGURE 19:**
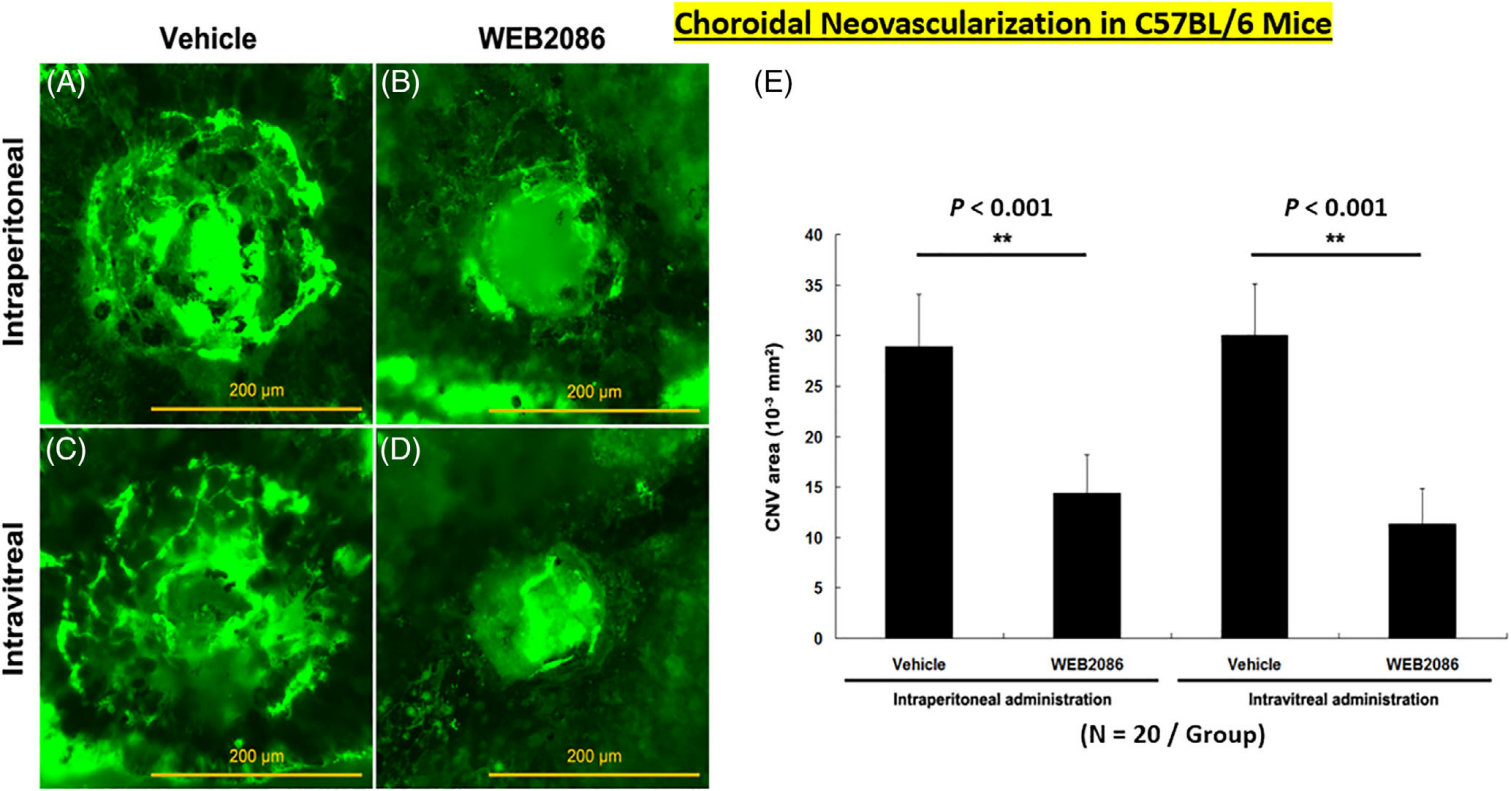
Fluoresceine angiographic monitoring of increased retinal vascular leakage (due to increased permeability)following laser‐induced choroidal neovascularization (CNV) in mice and the blockade of fluoresceine leakage by prior treatment with the PAF receptor antagonist, WEB2086 (intraperitoneal and intravitreal administration), is shown in the various figures. Adapted and modified with gratitude from Reference 92.

**FIGURE 20:**
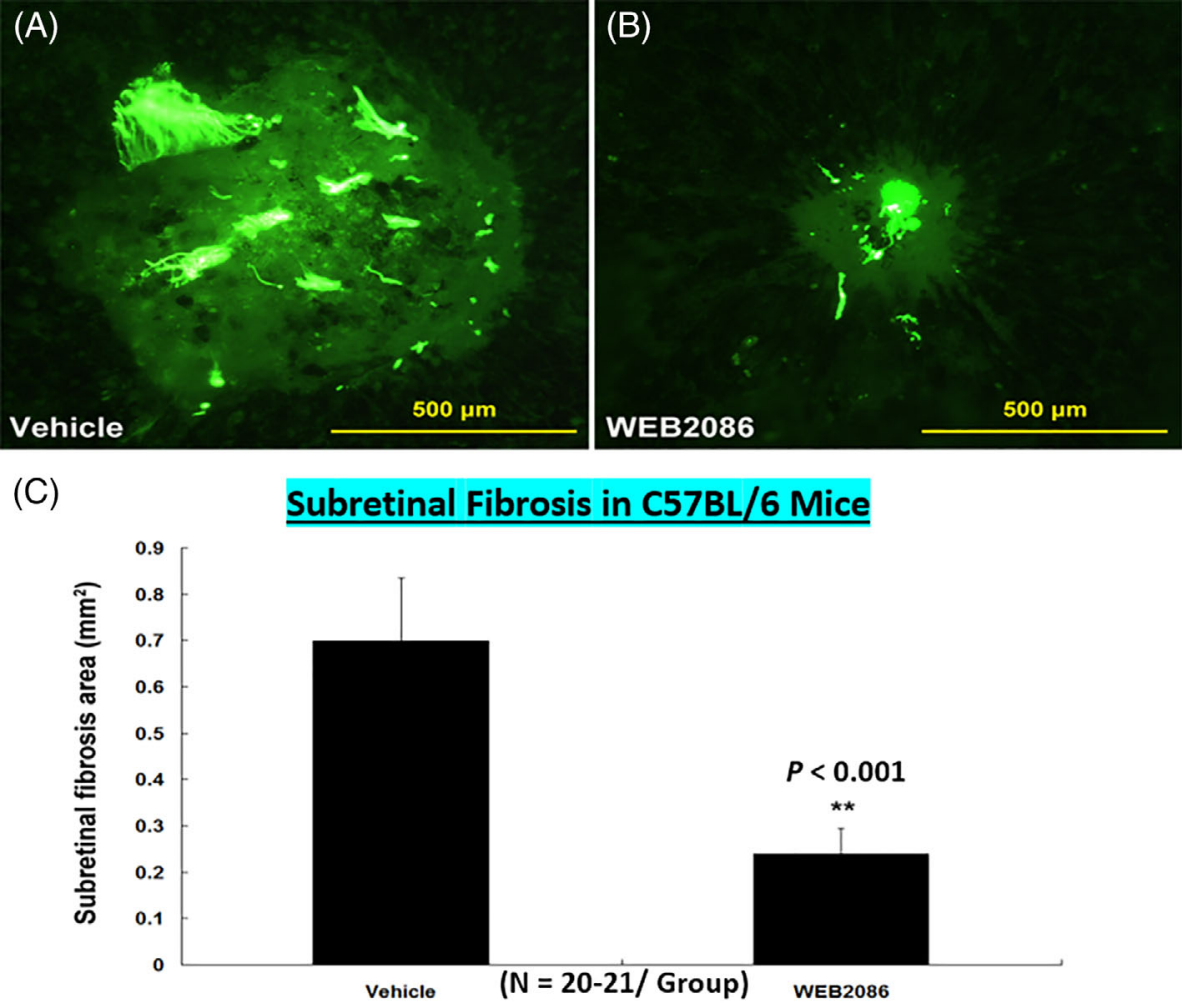
The reduction of sub‐retinal fibrosis by treating mice with the PAF receptor antagonist, WEB‐2086, is shown. Adapted and modified with gratitude from Reference 92.

### 4.2 PAF, uveitis, and ocular angiogenesis

As the term implies, uveitis is the inflammation of the uveal tract covering the ANC and the vitreoretinal areas of the eye and involves trauma‐, injury‐ or pathogen‐induced events involving the iris, ciliary body (CB) and the retina‐choroid.^80^, ^81^, ^82^, ^83^, ^84^, ^85^, ^86^, ^87^ Untreated uveitis is responsible for up to 20% of blindness since the inflammation causes development of cataracts, elevated intraocular pressure (IOP) and glaucomatous optic neuropathy, macula edema and potential retinal detachment due to development of edema at the back of the eye. Much evidence implicates PAF and its pre‐cursor/metabolite, Lyso‐PAF, in causing ocular inflammation relative to uveitis causation. PAF receptors present in the iris/CB^88^ activated by exogenous PAF promote arachidonic acid release which leads to formation of PGs^89^ in the eye which then increase vascular permeability and which contribute to cystoid macular edema.^86^ Additionally, PAF plays a role in chorioretinitis^90^, ^91^ and chorioretinal neovascularization^92^, ^93^, ^94^ which cause serious visual disability through induction of macular degeneration which effects central vision and acuity.^95^, ^96^

Copious amounts of PAF are released from iris/CB in endotoxin‐induced uveitis and it causes local inflammation.^12^, ^90^, ^97^ Similarly, exogenously injected PAF into the vitreous causes inflammation and chemotactically mobilizes leukocytes, responses that can be suppressed by PAF antagonists.^86^, ^90^, ^91^, ^97^ Furthermore, choroidal neovascularization and subretinal fibrosis induced by PAF is mediated through up‐regulation of angiogenic growth factors,^92^, ^94^ together with up‐regulated PAF‐receptor expression, and inhibited by PAF receptor antagonists such as WEB‐2086.^92^

Even though corticosteroids are used to currently treat uveitis, this is associated with many side‐effects that include increased IOP leading to glaucoma. Also, even though immunosuppression is also used as a remedy for this disorder, it causes enhanced vulnerability to opportunistic infectious agents. Likewise, while use of the anti‐TNF biologic, adalimumab, for the treatment of non‐infectious posterior, intermediate, and pan‐uveitis has met with some success, this is associated with systemic side‐effects and adverse events. Another emerging treatment option for non‐infectious uveitis involves intravitreally injected mTOR inhibitor, sirolimus.^87^

### 4.3 PAF and its role in retinal diseases

As with other tissues and cells, PAF at pico‐nano‐molar concentrations exerts important physiological functions in the retina being involved in neurotransmitter release and promoting dendritic/neurite elaboration. As mentioned above, chronically elevated levels of PAF signal retinal pathologies such a retino‐choroidal neovascularization and fibrosis^92^ (Figure 19) which could be significantly inhibited by blockade of PAF receptors in outer cell membrane and nuclear cell membranes of RPE and choroidal endothelial cells (Figure 20).^92^ In this context, during retinal ischemia, the oxidative stress triggers PAF production and secretion and vulnerable RPE and photoreceptor cells can also die from apoptosis.^98^, ^99^, ^100^, ^101^ Under these conditions, the PAF antagonist LAU0901 efficaciously protected photoreceptors and their terminals,^101^ and BN52021 effectively suppressed damage to rod outer segments in lithium‐ and light‐induced retinal degenerations animal models.^102^

In diabetic retinopathy, the hyperglycemia‐induced death of pericytes of retinal blood vessel capillaries and increased retinal vascular permeability causes retinal edema and can lead to proliferative diabetic retinopathy due to neovascularization.^103^, ^104^ Intravitreally injected PAF induced disruption of the choroidal endothelial cell layer and polymorphonuclear leukocyte infiltration which could be mostly abolished by pretreatment with the PAF antagonist BN52021 in animals.^97^ Furthermore, in streptozotocin‐induced diabetes in rats, the PAF antagonist WEB‐2086 helped preserve retinal blood vessel density.^26^ The latter observations were important also from the view‐point that PAF antagonists may be protective in retinopathy of prematurity (ROP) where retinal microvascular degeneration also occurs.^99^ Similarly, studies by Beauchamp et al.^105^ showed that retinal PAF concentration was elevated following a challenge with oxidative stress in vitro and also in rat pups' retinas when the animals were exposed to high levels of oxygen (model of ROP). This resulted in significant loss of retinal capillary endothelial cells which could be reduced by prior treatment with CV‐3988.^105^ Additional disease‐linkage studies showed that the expression of the PAF catabolic enzyme, PAF‐acetyl hydrolase (PAF‐AH), was significantly elevated in retinas of diabetic rats.^106^ And, this seemed to translate to clinical studies since plasma PAF‐AH was also up‐regulated in diabetic patients with proliferative retinopathy.^107^ However, whether these observations were causal or consequential of the disease remains to be determined. Nevertheless, PAF seems to be a contributor to the pathological milieu of factors and processes in retinal diseases and perhaps PAF receptor antagonist can have a role in mitigating some signs and symptoms associated with diabetic retinopathy, ROP and age‐related macular degeneration (AMD). However, in view of the success of anti‐VEGF biologics in treating the latter disorders,^96^ much more compelling clinical evidence is needed for PAF receptor antagonists to be progressed into development and for future approvals by health agencies. Given that maculopathy develops after long‐term anti‐VEGF treatment,^108^ perhaps it is time to consider PAF receptor antagonists as another treatment option for AMD. For this to happen, the PAF antagonist molecules would need to be hydrophobic enough to enter the affected cells in order to block the intracellular PAF receptors on the nuclear membranes. This is a critical physiochemical property^8^ to ensure that the gene expression of angiogenic factors, MMPs and additional inflammatory cytokines can be switched‐off and thus help abrogate the disease progression.^3^, ^4^, ^5^, ^6^, ^7^

## 5 ROLE OF PAF IN PAIN AND NEURODEGENERATION

The sensation of pain acutely signals danger and elicits an immediate response. Chronic pain, however, signals a disease‐causing malady requiring medicinal and/or other therapeutic intervention. One example is peripheral nerve injury leading to neuropathic pain. Purinergic receptor‐channel activation by ATP causes release of PAF and induces pain which could be blocked by the PAF antagonist ABT‐491, or by genetic deletion of the PAF synthesizing enzyme.^109^, ^110^, ^111^, ^112^ Since the cornea is extremely well innervated and highly sensitive to stimuli, the presence of lipid species related to platelet‐activating factor (PAF) and/or lyso‐phosphatidylcholine, phosphatidylcholine, and sphingomyelin in both the normal and dry eye rabbit tears,^113^ may be involved in causing ocular pain during injury, trauma, dry eye and in other conditions of ocular surface irritation as in SAC/PAC. Even though ocular itch is not directly linked to pain per se, the anti‐pruritic activity of PAF receptor antagonists (Figure 17),^51^ and the potential utility of such drugs in blocking dry eye‐induced pain is worth pursuing in the future.

Many studies have revealed that PAF is an important endogenous mediator of neuronal development, but becomes an inflammatory agent in the central nervous system (CNS) at acutely elevated and chronically high concentrations.^114^, ^115^ PAF is generated and released in response to cold injury,^116^ and it causes neuronal death via triggering Ca^2+^‐overloading of neurons due to glutamate receptor activation and nitric oxide (NO) production.^117^, ^118^ PAF is known to disrupt long‐term potentiation and thus memory consolidation,^119^ and PAF antagonists were able to block these deleterious effects of PAF in vitro in hippocampal neurons.^120^ Additional studies have described PAF antagonists affording neuroprotection against amyloid‐β‐induced neuronal apoptosis^100^, ^121^ and in cerebral ischemia‐induced cortical neuronal death.^122^ Likewise, cuprizone (CPZ)‐induced demyelinating model exhibited an up‐regulated profile of PAF‐receptor expression in astrocytes within several brain regions.^123^ Although Cheng et al.^124^ reported that elevated levels of PAF are associated with the onset of diabetic retinopathy and neurodegeneration, the literature is sparse on role of PAF in retinal neuronal degeneration and optic nerve structure/function. However, the presence of PAF receptor mRNA in retinal ganglion cells and retinal microglia^9^ strongly suggests that PAF has potential patho‐physiological roles in these cell types at the back of the eye. Likewise, since PAF induces functional responses in human trabecular meshwork cells in the AQH drainage system in the ANC,^125^ one wonders whether PAF has any role to play in AQH dynamics which are affected in the etiology of open‐angle and/or close‐angle glaucoma or in other types of glaucoma such as uveitic glaucoma.^126^ With such open questions remaining, further studies directed to ocular neurons and optic nerve and the effects of PAF in mediating glaucomatous optic neuropathy need to be conducted.

## 6 CELL‐MEMBRANE VS. NUCLEAR PAF RECEPTORS AND RECEPTOR‐INDEPENDENT EFFECTS OF PAF

Lastly, there has been a debate about whether all effects of PAF described in various cells, tissues and organs described above are mediated via its cell‐membrane‐bound receptors and on the relative roles of cell‐surface and nuclear‐membrane‐bound PAF receptors.^127^, ^128^, ^129^ Based on the schematic in Figure 3, it would appear that both sets of receptors are involved in many if not all physiological and pathological actions of PAF. It could be that the acute transmitter/mediator and physiological effects recruit only the outer cell‐membrane‐bound PAF receptors, whereas the nuclear receptors are involved in chronic and pathological conditions but also require the cell‐membrane‐bound PAF receptors. Alternative explanations may be that C16‐ and C‐18‐forms of PAF engage with different G‐proteins and exert their actions via differentially localized PAF‐receptors; perhaps PAF interacts with receptor sub‐types;^130^ and of course there is the distinct possibility that species differences^131^ in PAF receptors and their activation also influence the final outcomes in different tissues and under different conditions. The apparent receptor‐independence of some biological effects of PAF and Lyso‐PAF^132^, ^133^, ^134^, ^135^, ^136^, ^137^, ^138^, ^139^ could also be explained by the afore‐mentioned dichotomies. Clearly, all these aspects require further investigation and resolution. However, since specific antagonists of diverse chemical structures (Figure 4) block all the actions of PAF as described above, these pharmacological characteristics denote the involvement of specific receptors regardless of their localization in or on the cells being targeted.

## 7 CONCLUSIONS AND FUTURE PERSPECTIVES

PAF(C16‐ and C‐18 forms) is the most potent and efficacious lipid‐based constitutively generated phospholipid molecule that is derived from cell and nuclear membranes. It is active at sub‐picomolar concentrations and exerts its physiological and pathological roles by activating multiple signal transduction pathways using recruitment of different G‐proteins to mediate its effects. It can be generated de novo at low levels and can stay associated with the membranes until a stimulus promotes its mobilization and/or extrusion or regulated release as in mast cell degranulation. It can also be rapidly produced and secreted to signal injury, trauma or infection.

Due to its hydrophobic nature, PAF and related ether lipids can penetrate the cell and nuclear membranes and thus can affect cytoplasmic and nuclear genomic actions. At certain high concentrations it can bind to the receptors and desensitize them by being internalized as a PAF‐receptor complex. Whether this internalization process then culminates into subsequent release of active PAF into the cytoplasm is unknown. However, this is strongly indicated since PAF can bind to receptors on the nuclear membranes and trigger production of transcription factors, cytokine, proteolytic enzymes and growth factors. The origin of the cytoplasmic PAF may also be through activation of PLA2 and its action on nuclear membrane phospholipids (Figure 3).

As reviewed by Lordan et al.,^7^ fortunately there are a few examples of PAF receptor antagonists demonstrating modest efficacy in asthma patients (SR27417A), and in terms of improvement of bronchial hyperresponsiveness (Y‐24180), and reduction of PAF levels (BN‐52021) in patients. Additional efficacy of PAF antagonists (WEB‐2086) has been noted in the reduction of erythema induced by UV‐light, and reduction of organ dysfunction and morbidity linked to septic shock (TCV‐309). While rupatadine exhibited beneficial effects in chronic idiopathic urticaria and allergic rhino‐conjunctivitis, this probably represents synergistic activity due to blockade of HIS and PAF receptors. However, this also requires further studies to delineate the roles of both these receptor types in mediating the latter disease processes.

Another remaining enigma is associated with the relatively lack of efficacy of various PAF receptor antagonists in multiple human chronic diseases such as ulcerative colitis, myocardiac infarction, organ failure associated with pancreatitis, cognitive decline resulting from coronary artery bypass graft surgeries, psoriasis, etc. (reviewed in Reference 7). However, their inactivity could be explained, at least partially, by the fact that the PAF antagonists used (Lexipafant, Modipafant, RO‐24‐238 and SR27417A) were unable to enter the cell interior of affected tissues to prevent PAF's nuclear transcriptional actions. Nevertheless, this represents a surmountable challenge and much more effort needs to be expended to solve the physiochemical problems related to PAF antagonists.^8^ The poor translatability of animal model‐derived results with PAF and its inhibitors to clinical outcomes for patients has also puzzled researchers. Perhaps the optimal route(s) of administration of PAF antagonists need to be determined to permit them to reach their site of action and to maintain a high enough local concentration to achieve efficacy, and this may include sublingual therapy.^140^, ^141^ However, while the several failures of PAF receptor antagonism to mitigate human disease development and progression elaborated by PAF are disappointing, design and synthesis of novel hydrophobic molecules that could block PAF's intracellular actions may lead to future successes.^8^ The elucidation of the precise role of nuclear PAF receptors^92^, ^142^, ^143^ and orphan receptors,^144^ and receptor dimers^145^ that may recognize PAF and Lyso‐PAF as their cognate ligands is also needed. We eagerly await more progress in the PAF receptor mechanisms of action and the creation of next generation of PAF receptor antagonists,^8^ perhaps those that can be conjugated to other disease‐modifying drugs, siRNAs and/or antibodies.^146^, ^147^, ^148^, ^149^ In fact, PAF‐neutralizing antibodies may prove effective in some of the diseases caused by PAF akin to what has been achieved by anti‐VEGF biologics,^150^, ^151^, ^152^ and more recently by use of bispecific antibodies.^153^ Exciting new technologies and other forms of therapeutic agents as mentioned above need to be applied to PAF and its receptors to help advance this field of study.

## CONFLICT OF INTEREST

The author declares no conflict of interest and only wishes to gather, assemble and disseminate timely and relevant information on the subject matter to help students and researchers in their endeavors to learn and seek suitable remedies for the eye diseases where PAF is implicated.

**Abbreviations**
AC: allergic conjunctivitis
ANC: anterior chamber
AQH: aqueous humor
AMD: age‐related macular degeneration
BK: bradykinin
CB: ciliary body
CFs: corneal fibroblasts
CEC: conjunctival epithelial cells
CEPI: corneal epithelial cells
CNS: central nervous system
CPZ: cuprizone
HIS: histamine
IOP: intraocular pressure
HCEC: human conjunctival epithelial cells
LT: leukotrienes
MMPS: matrix metalloproteinases
PGs: prostaglandins
PAC: perennial allergic conjunctivitis
PLC: phospholipase C
RPE: retinal pigmental epithelium
SAC: seasonal allergic conjunctivitis
TNFα: tumor necrosis factor‐α
VEGF: vascular epithelial growth factor
WHO: world health organization

## Data Availability

This review article is based on the literature searches conducted via PubMed search engine until February 2022. It covers many important references that have impacted the PAF receptor pharmacology and actions in various bodily functions and diseases, especially in the eye and associated visual system. The author apologies if some key citations have been inadvertently omitted.
